# New Acetamide-Sulfonamide-Containing Scaffolds: Antiurease Activity Screening, Structure-Activity Relationship, Kinetics Mechanism, Molecular Docking, and MD Simulation Studies

**DOI:** 10.3390/molecules28145389

**Published:** 2023-07-13

**Authors:** Saghir Ahmad, Muhammad Abdul Qadir, Mahmood Ahmed, Muhammad Imran, Numan Yousaf, Tanveer A. Wani, Seema Zargar, Ijaz Ali, Muhammad Muddassar

**Affiliations:** 1 School of Chemistry, University of the Punjab, Lahore 54590, Pakistan; saghirtalib@gmail.com (S.A.); mabdulqadir@gmail.com (M.A.Q.); 2 Department of Microbiology, Immunology and Cancer Biology, School of Medicine, University of Virginia, Charlottesville, VA 22904, USA; 3 Department of Chemistry, Division of Science and Technology, University of Education, College Road, Lahore 54770, Pakistan; 4 Kauser Abdulla Malik School of Life Sciences, Forman Christian College (A Chartered University), Lahore 54600, Pakistan; muhammadimran@fccollege.edu.pk; 5 Department of Biosciences, COMSATS University Islamabad, Park Road, Islamabad 45550, Pakistan; numanyousaf427@gmail.com; 6 Department of Pharmaceutical Chemistry, College of Pharmacy, King Saud University, P.O. Box 2457, Riyadh 11451, Saudi Arabia; twani@ksu.edu.sa; 7 Department of Biochemistry, College of Science, King Saud University, P.O. Box 222452, Riyadh 11451, Saudi Arabia; szargar@ksu.edu.sa; 8 Center for Applied Mathematics and Bioinformatics, Gulf University for Science and Technology, Mubarak Al-Abdullah 32093, Kuwait; ali.i@gust.edu.pk

**Keywords:** sulfonamides, NSAIDs, urease, kinetics studies, drug likeness, in silico studies

## Abstract

The development of novel scaffolds that can increase the effectiveness, safety, and convenience of medication therapy using drug conjugates is a promising strategy. As a result, drug conjugates are an active area of research and development in medicinal chemistry. This research demonstrates acetamide–sulfonamide scaffold preparation after conjugation of ibuprofen and flurbiprofen with sulfa drugs, and these scaffolds were then screened for urease inhibition. The newly designed conjugates were confirmed by spectroscopic techniques such as IR, 1HNMR, 13CNMR, and elemental analysis. Ibuprofen conjugated with sulfathiazole, flurbiprofen conjugated with sulfadiazine, and sulfamethoxazole were found to be potent and demonstrated a competitive mode of urease inhibition, with IC50 (µM) values of 9.95 ± 0.14, 16.74 ± 0.23, and 13.39 ± 0.11, respectively, and urease inhibition of 90.6, 84.1, and 86.1% respectively. Ibuprofen conjugated with sulfanilamide, sulfamerazine, and sulfacetamide, whereas flurbiprofen conjugated with sulfamerazine, and sulfacetamide exhibited a mixed mode of urease inhibition. Moreover, through molecular docking experiments, the urease receptor-binding mechanisms of competitive inhibitors were anticipated, and stability analysis through MD simulations showed that these compounds made stable complexes with the respective targets and that no conformational changes occurred during the simulation. The findings demonstrate that conjugates of approved therapeutic molecules may result in the development of novel classes of pharmacological agents for the treatment of various pathological conditions involving the urease enzyme.

## 1. Introduction

A metal (Ni)-containing enzyme called urea amidohydrolase, commonly known as urease, catalyzes the hydrolysis of urea to produce ammonia and carbon dioxide [1]. Based on the information given, it may be inferred that all ureases share a common ancestor gene. Jack bean urease was the first to be extensively described, crystallized, and marked as a template for the characterization and investigation of the urease enzymes, and, as shown by the available data, there is no difference between the sequences of plants and bacteria, and both have the same active sites [2]. The development of efficient and secure urease inhibitors has been a key focus of pharmacological investigations due to the involvement of ureases in a number of pathological illnesses. Continuous ammonia production increases the permeability of the gastric mucosa, which leads to inflammation, ulcers, adenocarcinoma, and lymphoma [3,4]. Targeting urease activity can help eradicate *Helicobacter pylori* (*H. pylori*) in its early stages of infection because the bacterium depends on it for survival in the stomach’s low pH environment [5,6]. In order to cure diseases caused by enzyme disorders, enzyme inhibitors are used. Inhibitors are the molecules that can disrupt enzymatic bioactivity by binding themselves to the active site of the enzyme permanently or temporarily. In fact, they block the active sites of the enzymes and cease the enzyme-catalyzed biological reaction [7]. Enzyme inhibitors are present in nature and are also designed and produced as drugs. Most toxins present in nature are natural enzyme inhibitors. Synthetic enzyme inhibitors find application in treating diseases while acting as a drug. For the last few decades, the search for effective and safe urease inhibitors has been a challenge for many scientists in the pharmaceutical industry. Since urease is linked to bacterial infections and there are currently very few urease inhibitors available, the design and synthesis of novel urease inhibitors is our prime research interest [8,9,10].

**Figure 1:**
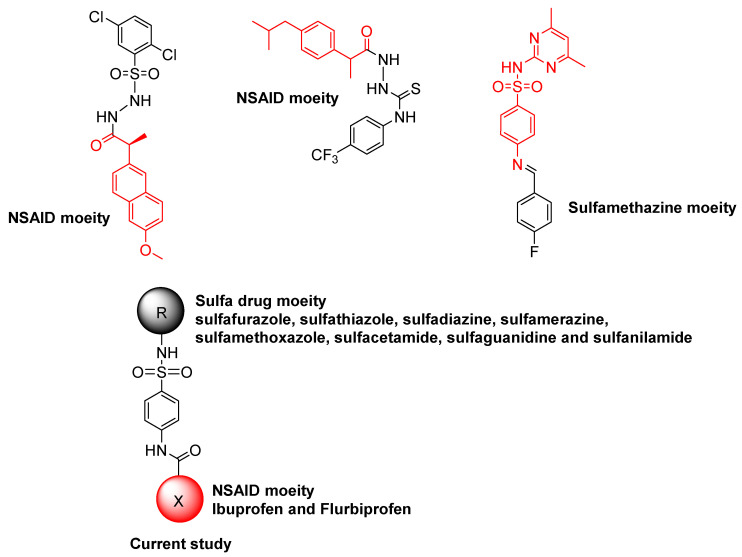
Sulfa drugs and NSAID-derived compounds as urease inhibitors.

Sulfonamides, also called sulfa drugs, have -SO_2_NH- moiety and are derived from the sulfonic acid group (RSO_3_H) from their reaction with an amino group, which replaces the hydroxyl group, forming sulfonamides. As a sulfonyl group is a constituent of different biological active molecules, sulfonamides show a wide range of biological activities, which secure their unique position in the pharmaceutical drug industry as well as in medicinal chemistry [11,12]. The various biological activities exhibited by sulfonamides include diuretic, anti-thyroid, hypoglycemic, anti-conversant, anti-bacterial, anti-hypersensitive, protease inhibition, anti-diabetic, anti-carbonic anhydrase, anti-urease, anti-migraine, anti-fungal, anti-inflammatory, and herbicidal activities [13,14,15,16,17,18]. In the field of medicinal chemistry, acetamide moiety finds many applications in clinical prescription drugs due to its high therapeutic potential for targeting various disease models, such as treatment of infections, convulsions, and allergies. Acetamide-containing moiety-containing drugs are also used for other ailments, such as pain and inflammation control (nepafenac, ibuproxam), COX enzyme inhibition (bufexamac), and as antiviral (oseltamivir) drugs [19,20,21]. To create effective and secure urease inhibitors, numerous sulfonamide/acetamide derivatives, including those of already-marketed medications, have undergone extensive research in recent years (Figure 1). The results of this study revealed a number of drug-based compounds that can be used as leading candidates for the continued development of innovative, highly effective urease inhibitors [6,22]. Diverse biological activities, such as α-amylase, β-glucuronidase, α-glucosidase, and urease inhibition, were explored by molecules derived from non-steroidal anti-inflammatory drugs (NSAIDs), including ibuprofen, naproxen, flurbiprofen, and piroxicam [6,7,23,24]. 

We set out to design and synthesize a number of conjugates of NSAIDs and sulfa medicines, making use of the multi-target strategy that is attracting increasing interest among pharmaceutical chemists worldwide. The synthesis of conjugates has been an important tool and is aimed at modifying the action of existing drugs, particularly to reduce side effects and to potentiate the action. It is known from the literature that more than 60% of existing drugs are derivatives of known molecules. Therefore, in this context, new conjugates were developed by coupling NSAIDs (ibuprofen and flurbiprofen) and sulfa drugs (sulfanilamide, sulfisoxazole, sulfathiazole, sulfadiazine, sulfamerazine, sulfamethoxazole, sulfacetamide, and sulfaguanidine) to perform an amide-coupling reaction (in situ). The newly developed conjugates contained the acetamide–sulfonamide scaffolds and were screened for urease inhibition. The newly synthesized conjugates were mechanism-based inhibitors that bound in the active site through electrostatic interactions with Ni ions and hydrogen bonds with neighboring residues. Our design strategy is built on retaining the pharmacophoric moiety in our target molecules. Additionally, in silico investigations were carried out to examine the function of the most potent inhibitors as ligands against the urease enzyme by molecular docking studies. The stability of most active inhibitors with said enzymes was also confirmed by analyzing the MD trajectories generated by 50 ns simulation. 

## 2. Results and Discussion

### 2.1. Chemistry

**Scheme 1:**
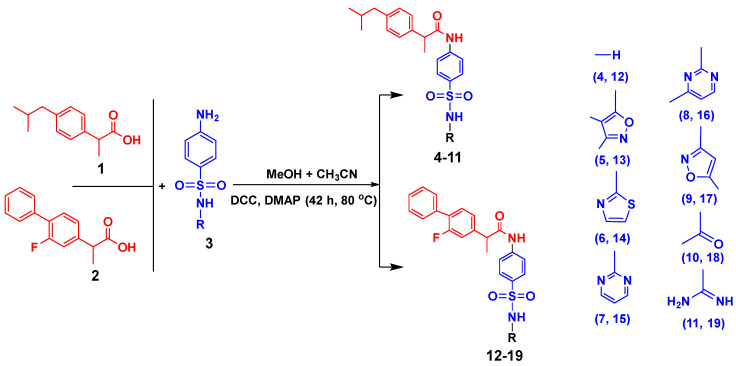
Synthesis procedure for conjugates of sulfa drugs with ibuprofen and flurbiprofen.

The synthesis of target conjugates (Scheme 1) was performed by coupling ibuprofen and flurbiprofen with sulfa drugs (sulfanilamide, sulfisoxazole, sulfathiazole, sulfadiazine, sulfamerazine, sulfamethoxazole, sulfacetamide, and sulfaguanidine). For the coupling of the amino group of the sulfa drugs with the hydroxyl of the NSAIDs (ibuprofen and flurbiprofen), DCC was used as a coupling agent in the presence of a DMAP catalyst. A number of investigations have revealed that the most widely employed reaction in medicinal chemistry is, in fact, amide coupling. The synthesis of a huge variety of compounds is possible due to a well-known reaction between two readily available synthons: a carboxylic acid and an amine. In recent years, sulfonamide linkers have become more widely used in medicinal chemistry [25]. The details of the synthesis are presented in the experimental section. The newly synthesized conjugates were characterized by spectroscopic techniques (^1^H-NMR, ^13^C-NMR, and IR), and elemental analysis; the details are presented in Section 3.1.3.

In the IR spectrum of conjugates, the -NH moiety present in sulfonamides showed the absorption band at 3458–3465 cm^−1^. The absorption bands at 2919–2926 cm^−1^ represented the -NH of the acetamide moiety in the compounds. The -NH-S=O group showed absorption bands in the 1359–1366 cm^−1^ (unsymmetrical) and 1138–1142 cm^−1^ (symmetrical) regions. In the IR spectra of all newly synthesized conjugates, the presence of an -NH-S=O group was confirmed by the absorption band appearing in the region of 1021–1031 cm^−1^. In the proton NMR (^1^HMR) spectra of the conjugates, the peaks appearing at δ 10.06–11.1 ppm exhibited the presence of an -NH proton of the acetamide group, confirming the coupling of both drugs. The singlet at δ 1.78–1.98 ppm showed the presence of a proton of the methyl group (-CH_3_) attached to the acetyl group. The values of chemical shifts and integrals of all remaining aromatic and aliphatic protons were already mentioned in Section 3.1.2. The peaks at 170.3–176.4 ppm in the ^13^C NMR spectra showed the presence of a carbonyl carbon group present in the compounds. In all compounds, the chemical shift signal at δ 24.2–26.2 ppm demonstrated the presence of acetyl carbon. The spectral ^13^C NMR analysis of all compounds with assigned structures was consistent (Supplementary Materials, Figures S1–S48).

### 2.2. Pharmacological Activity

#### 2.2.1. Urease Inhibition and Structure–Activity Relationship (SAR)

We evaluated the conjugates that were successfully synthesized for their in vitro anti-urease action. In the urease inhibition investigations, thiourea served as a reference and exhibited an IC_50_ value of 22.61 µM. Table 1 presents the enzyme (urease) inhibition data, and all of the conjugates were effective against it. 

**Scheme 2:**
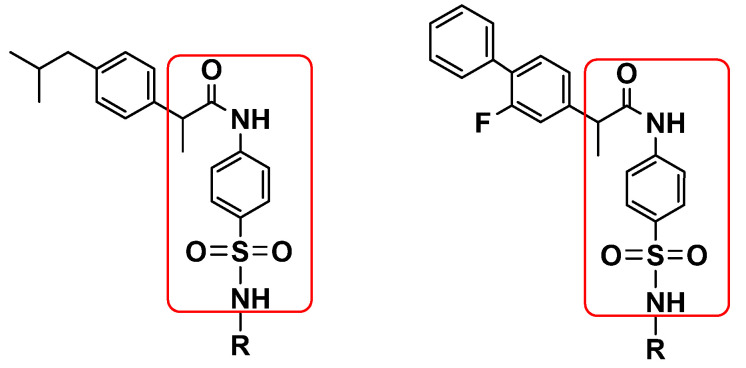
Illustration of SAR studies.

SAR studies (Scheme 2) were carried out purely based on the central core containing acetamide and a substituted sulfonamide scaffold. The target molecules were split into two groups in order to rationalize the findings: One was ibuprofen and other was flurbiprofen, which were coupled with various substituted sulfonamides (sulfa drugs). The effective structural feature of the most active inhibitor (90.6% inhibition) comprised a thiazole-substituted sulfonamide with ibuprofen (**6**, IC_50_ = 9.95 ± 0.14 µM), whereas the same substituted sulfonamide with flurbiprofen (**14**, IC_50_ = 63.42 ± 1.15 µM) showed six-fold less activity against the urease (60.4% inhibition). It is evident that the acetamide linked to phenyl-alkyl groups showed better activity than the fluoro-substituted biphenyl group. Acetamide–sulfonamide scaffolds containing methyl-diazine and acetyl substitution (**8**, **10**, **16**, and **18**) on the sulfonamide side showed almost the same urease inhibition activity regardless of the phenyl-alkyl and fluoro-substituted biphenyl group on the acetamide side. Furthermore, isoxazole and diazine substituted on the sulfonamide side and the fluoro-substituted biphenyl group on the acetamide side showed better urease inhibition activity compared to the phenyl-alkyl group on the acetamide side (Table 1).

#### 2.2.2. Enzyme Kinetics Studies 

Ibuprofen conjugated with sulfathiazole (**6**), flurbiprofen conjugated with sulfadiazine (**15**), and sulfamethoxazole (**17**) were found to be potent and showed a competitive mode of urease inhibition, with IC_50_ (µM) values of 9.95 ± 0.14, 16.74 ± 0.23, and 13.39 ± 0.11, respectively, and urease inhibition of 90.6%, 84.1%, and 86.1% respectively.

**Scheme 3:**
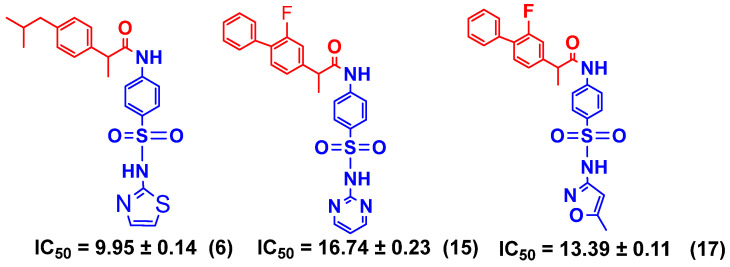
Illustration of competitive inhibitors.

The competitive mode of inhibition of conjugates (**6**, **15**, and **17**, Scheme 3) was demonstrated by kinetics studies. The kinetics studies were performed by using five different concentrations (0.0, 5.0, 10.0, 15.0, and 20.0 µM) of each conjugate while using four different concentrations of urea (0.5, 1.0, 2.0, and 4.0) as substrate. 

**Figure 2:**
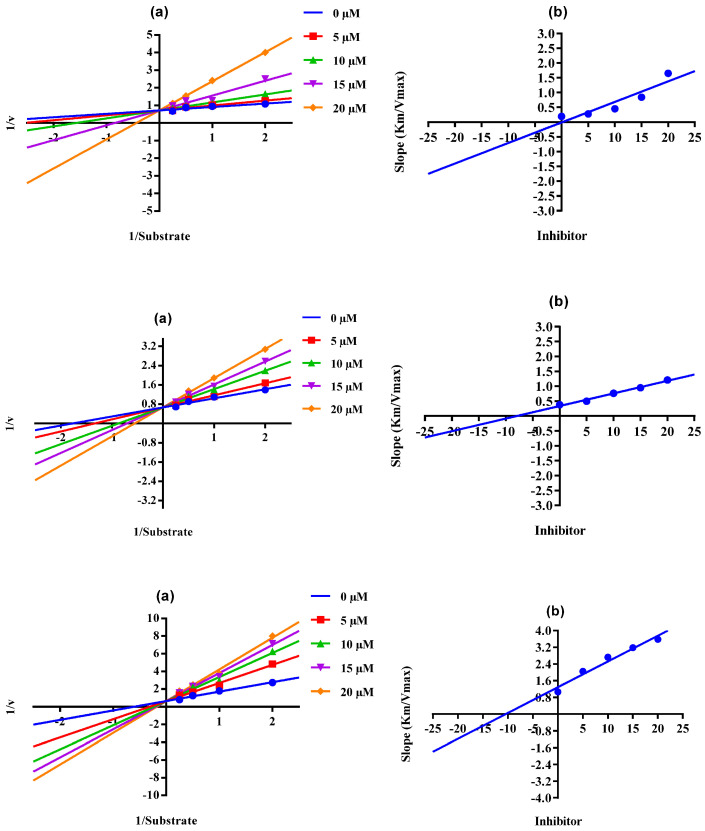
Demonstration of the competitive mode of the inhibition of conjugates **6**, **15**, and **17** by kinetics studies. (**a**) Primary Lineweaver–Burk plot for K_m_- and V_max_-value calculation, (**b**) secondary Lineweaver–Burk plot for K_i_-value calculation.

The Lineweaver–Burk plot is a powerful tool for analyzing enzyme kinetics and determining the mode of inhibition of an enzyme by a particular conjugate. The inhibitor molecule (conjugate) attaches to the enzyme’s active site in competitive inhibition and prevents the substrate from attaching. In a Lineweaver–Burk plot, competitive inhibition is characterized by a change in the slope of the line (K_m_, also called the Michaelis constant), whereas the intercept remains the same (V_max_, the maximum rate). The increase in the K_m_ value of the urease enzyme despite the value of V_max_ remaining constant at 20 µM of inhibitor concentration demonstrates that conjugates **6**, **15**, and **17** inhibited the enzyme in a competitive way (Figure 2). The inhibition constant (K_i_) value of each conjugate was also calculated by plotting the slope of each line vs. different concentrations of each conjugate, also called secondary Lineweaver–Burk plots. The K_i_ value of conjugates **6**, **15**, and **17** were discovered to be 0.17, 7.95, and 10.40 µM, respectively (Table 1). The plots of the enzymatic kinetics of the competitive inhibitors are presented below in Figure 2. 

**Scheme 4:**
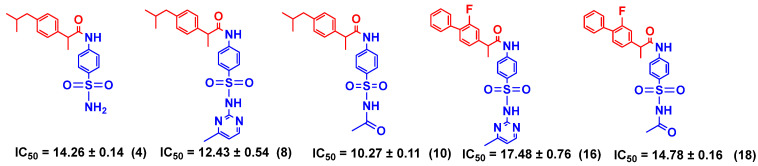
Illustration of mixed inhibitors.

**Figure 3:**
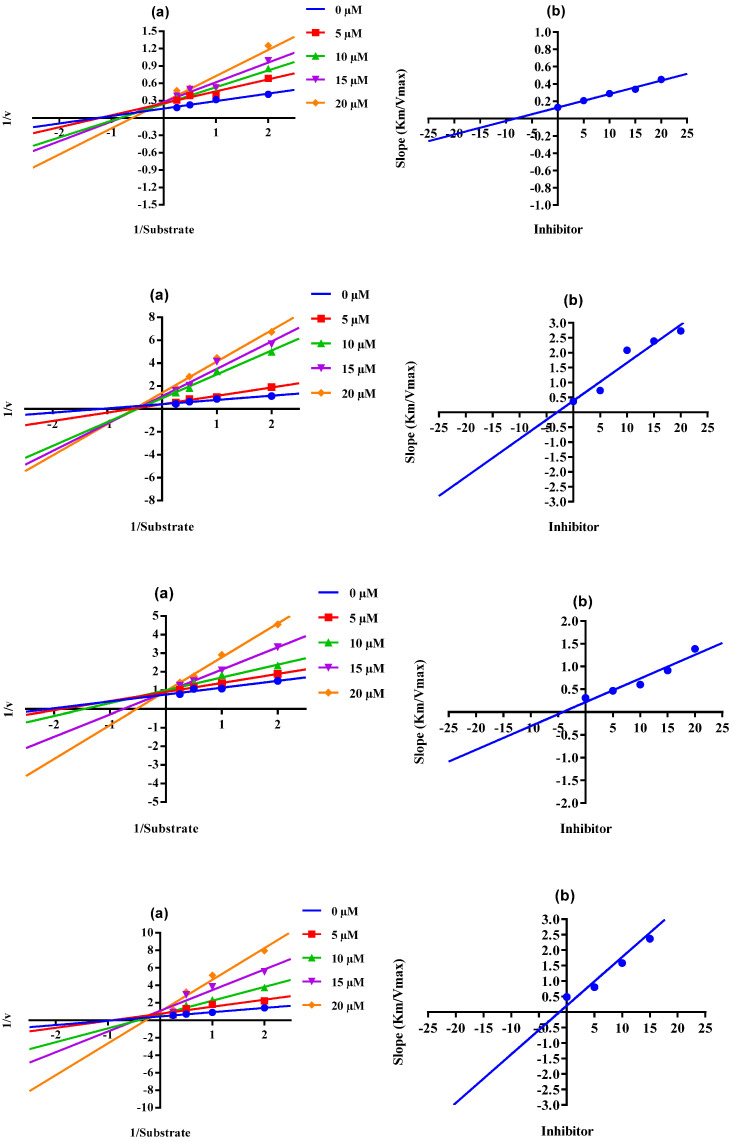

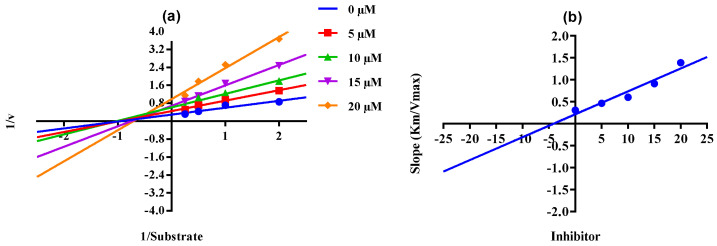
Demonstration of the mixed mode of the inhibition of conjugates **4**, **8**, **10**, **16**, and **18** by kinetics studies. (**a**) Primary Lineweaver–Burk plot for K_m_- and V_max_-value calculation, (**b**) secondary Lineweaver–Burk plot for K_i_-value calculation.

When there is mixed-type inhibition, the enzyme–substrate complex is bound by the inhibitor molecule, which prevents the reaction from happening, and increasing the substrate concentration will not remove this kind of inhibition. In a Lineweaver–Burk plot, mixed-type inhibition is characterized by a change in both the slope (K_m_) and the intercept of the line (V_max_). The mixed type of inhibition of the conjugates (**4**, **8**, **10**, **16**, and **18**, Scheme 4) was also demonstrated after the kinetics studies by using five different concentrations (0.0, 5.0, 10.0, 15.0, and 20.0 µM) of each conjugate while using four different conditions of urea (0.5, 1.0, 2.0, and 4.0) as substrate. The increase in the K_m_ value of the urease enzyme despite the value of V_max_ decreasing at 20 µM of inhibitor concentration demonstrates that conjugates **4**, **8**, **10**, **16**, and **18** inhibited the enzyme inhibition in a mixed way (Figure 3). The K_i_ values calculated from the secondary Lineweaver–Burk plots were 8.20, 3.01, 2.62, 1.30, and 4.11 µM (Table 1) for conjugates **4**, **8**, **10**, **16**, and **18**, respectively. The plots of the enzymatic kinetics of the mixed inhibitors are presented below in Figure 3. The rest of the conjugates (**5**, **12**, **13**, and **19**) also showed good inhibition for the urease in the range of 82.1–84.6% and IC_50_ values in the range of 23.27 ± 0.41–35.30 ± 0.21 µM. On the other hand, conjugates **7**, **9**, **11**, and **14** exhibited moderate inhibition of urease.

### 2.3. Molecular Docking and Dynamics Simulation Studies

**Figure 4:**
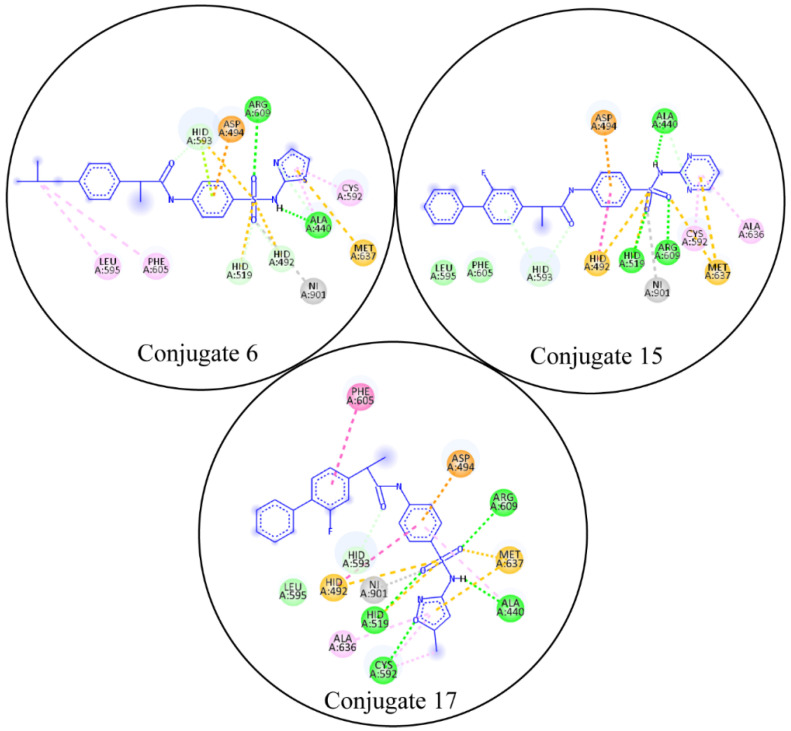
The molecular interactions of competitive inhibitors with urease. In the molecular interactions, the green spheres show hydrogen bonds, purple spheres show sigma bonds, orange spheres show pi-sulfur bonds, and magenta spheres show pi-alkyl bonds.

**Figure 5:**
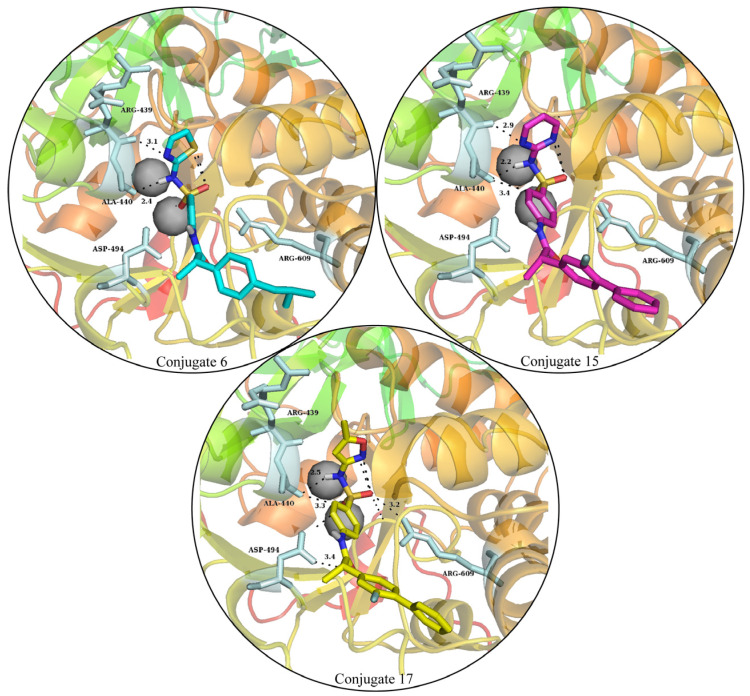
The plausible binding modes of competitive inhibitors in the urease-binding pocket. The hydrogen bonds are denoted with black dashes, with the optimum distance between the bond making atoms.

Through molecular docking, the competing inhibitors’ likely binding mechanisms were predicted and the molecular interactions were analyzed. In docking studies, it was observed that conjugate 6 made three hydrogen bonds with Ala440, His593, and Arg609; made one pi-sulfur bond with Met637; and had three hydrophobic interactions. Conjugate 15 was involved in hydrogen bonding with Ala440, His519, and Arg609; made two pi-sulfur bonds with His492 and Met637; and had van der Waals interactions with Leu595 and Phe605. Lastly, conjugate 17 made four hydrogen bonds with Ala440, His519, Cys592, Arg609; two pi-sulfur bonds; and four pi-alkyl bonds. The molecular interactions of the conjugate compounds are shown in Figure 4. The plausible binding modes were also visualized and are shown in Figure 5.

**Figure 6:**
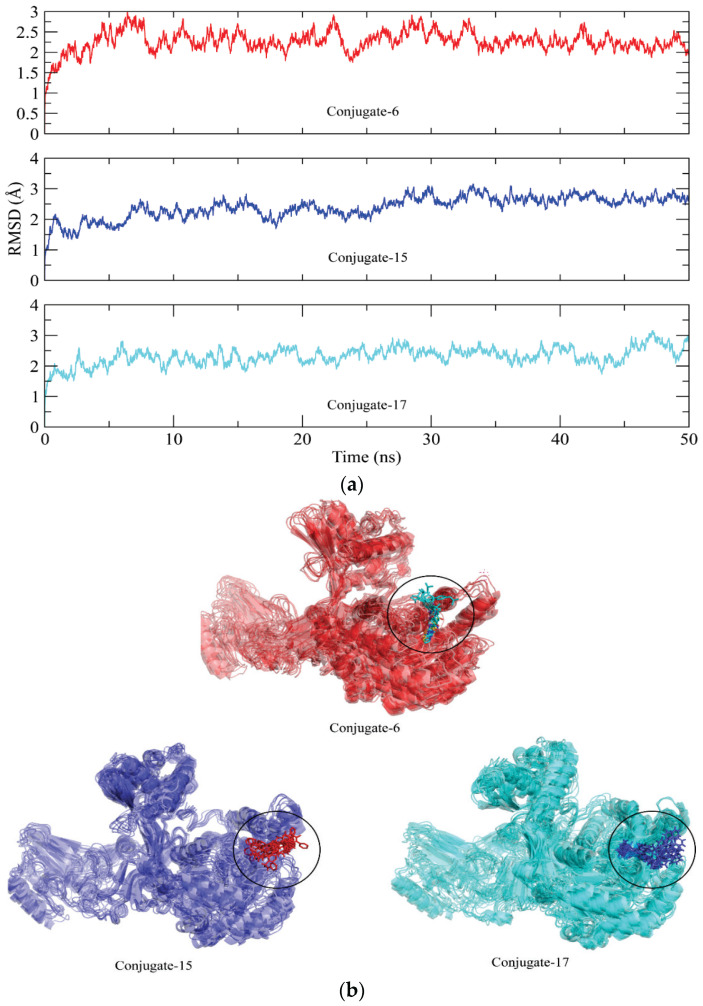
(**a**) Root mean square deviation plots of the urease backbone atoms calculated during 50 ns simulation. (**b**) The alignment of different snapshots of complexes obtained from the md trajectories.

The stability of the protein–ligand complexes was assessed using various molecular dynamics simulation analyses. The backbone atom RMSD of urease complexed with the conjugates was calculated to observe the structural stability of the complexes (Figure 6a) [26]. It can be observed that all complexes of urease equilibrated at 5 ns and the RMSD of the conjugate 6 complex showed deviations in the range of ~2–3 Å up to 30 ns. The RMSD attained stability after 30 ns and remained in the range of ~2.5 Å until the end of the simulation. On the other hand, the RMSD of the conjugate 15 complex maintained values in the range of ~2–3 Å throughout the simulation. Lastly, the RMSD of the conjugate 17 complex also indicated that the protein did not show deviations during the simulation and stayed in the ~2–3 Å range throughout the simulation. The minor deviations in the trajectories indicate the stability of the urease complexes. Furthermore, the binding stability of the complexes was analyzed by aligning the different snapshots of the md trajectories, and it was demonstrated that the ligands remained closely bound to the protein during simulation (Figure 6b).

**Figure 7:**
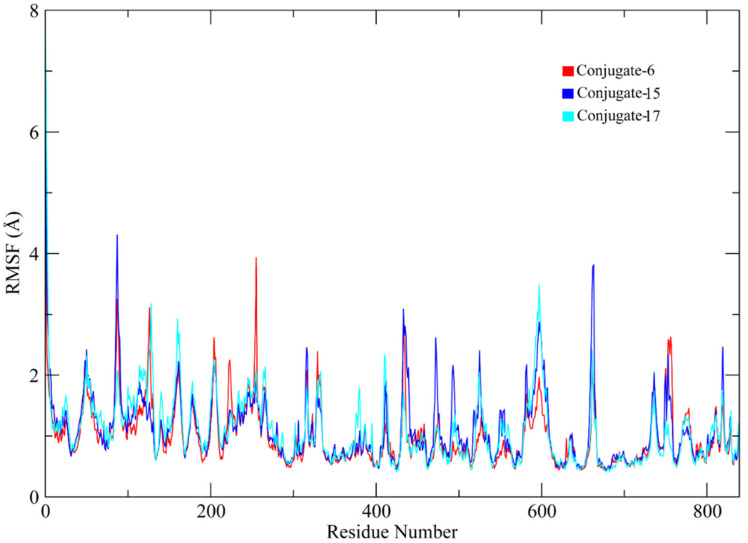
RMSF plots of the urease protein residues.

Residues at 100, 110 to 125, 260 to 270, 420 to 430, 590 to 610, and 630 to 640 showed major fluctuations, indicating the presence of loops. Other portions of the protein remained rigid during the simulation, except for the conjugate 15 complex, which showed major fluctuation in residues 90 to 100 compared to other complexes (Figure 7).

**Figure 8:**
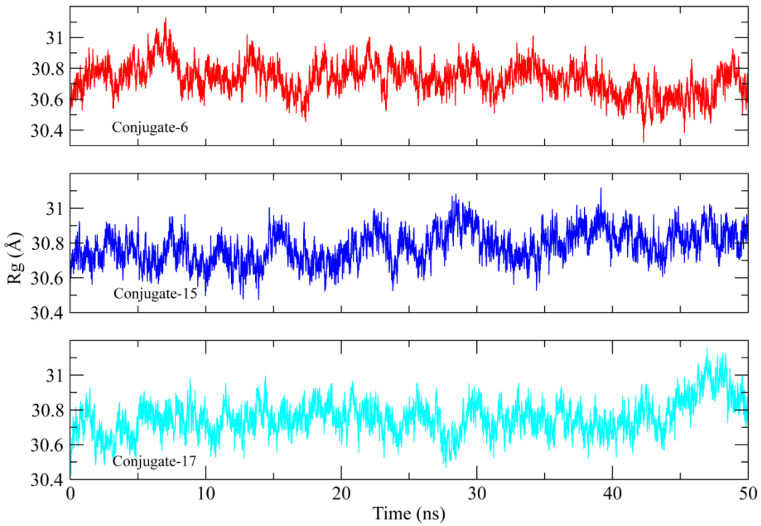
The radius of gyration calculation to analyze the compactness of the urease protein structure during the simulation.

Radius of gyration (Rg) analysis was performed to assess the structural compactness of the urease proteins when bound to the conjugate [27]. Lower Rg values indicate structural stability, whereas higher Rg values indicate that there were distortions in the structure during simulation. The Rg plots of the complexes showed that the Rg values maintained a range of ~30.06 to 31 Å after being equilibrated at 5 ns. The Rg value of conjugate 17 increased to ~31.04 Å after 45 ns. The stable Rg values indicate that the protein structures remained compacted during simulation when bound to these conjugates (Figure 8). 

The process of discovering new drugs is fascinating and cannot be approached or dealt with by a single tactic. The researchers concentrated their efforts on the repurposing strategy, which is essentially centered on finding new applications for well-known medications. We advanced in our present medicinal chemistry research and suggested manipulating recognized medications. We picked the sulfonamidation reaction as a proof-of-concept since sulfonamides are found in many well-known medications. As for the drugs, we chose sulfonamides and the carboxylic acid group containing NSAIDs, and the conjugation of these drugs resulted in acetamide, which is a motif that is present in several drugs. Additionally, the Lipinski rules verified all of the compounds (Table 2). It has been noted that orally active medications often only have five hydrogen bond donors (HBD) and no more than ten hydrogen bond acceptors (HBA). In addition, the values for the logP and molecular mass should be below 5 and 500 g/mol, respectively. Drug permeability is thought to be significantly influenced by hydrogen-bonding capability. Compounds with HBA and HBD readings above these values have been demonstrated to exhibit poor penetration. According to our findings, every synthetic conjugate followed the Lipinski rules. For the blockage of HERG K^+^ channels, the predicted IC_50_ value was less than −5, and the cell permeability in nm/sec to predict Caco2 cell permeability was in the recommended range (QPCaco2 = <25 poor, >500 great). All of the conjugates complied with the predicted brain/blood partition coefficient (QPlogBB = −3.0–1.2) and binding to human serum albumin (QPlogKhsa = −1.5–1.5) [28].

## 3. Materials and Methods

### 3.1. Chemistry

#### 3.1.1. General

Newly formulated conjugates were synthesized by utilizing high-purity sulfa drugs (sulfanilamide, sulfisoxazole, sulfathiazole, sulfadiazine, sulfamerazine, sulfamethoxazole, sulfacetamide, and sulfaguanidine) made by Sigma Aldrich, St. Louis, MO, USA, and purchased from Falcon Scientific, Lahore, Pakistan. NSAIDs (ibuprofen and flurbiprofen) were kindly gifted by Novamed Pharmaceuticals, Lahore, Pakistan. Conjugate structures were elucidated through spectral investigations using techniques including FTIR, ^1^HNMR-500 MHz, and ^13^CNMR-125 MHz (Bruker, Billerica, MA, USA). An HT+ elemental analyzer from thermos Scientific, UK, was used for the analysis of the elements (C, H, N, and S). To verify the purity of the synthesized conjugates, TLC examination was carried out using pre-coated silica plates from Merck, Germany, under UV light, and a Gallenkamp apparatus was used to find the melting point. 

#### 3.1.2. Synthesis Protocol for New Conjugates 

In a 100 mL flask, ibuprofen (1 mmol) was dissolved in solvent containing a mixture of methanol and acetonitrile in a 50:50 ratio. Then, 1 mmol of N, N′-dicyclohexylcarbodiimide (DCC) was added to the solution and the reaction was carried out with the addition of 4-dimethylaminopyridine (DMAP) as a catalyst. The reaction of ibuprofen and DCC continued for 30 min at 80 °C. Then, for amide bond formation, the respective sulfa drug, such as sulfanilamide, sulfisoxazole, sulfathiazole, sulfadiazine, sulfamerazine, sulfamethoxazole, sulfacetamide, or sulfaguanidine (1 mmol), was added to the reaction mixture. Refluxing of the reaction mixture was continued for 42 h to complete the reaction, and after amide bond formation, dicyclohexylurea (DCU) became precipitated. The TLC was run to monitor the progress of the reaction using ethyl acetate: methanol: n-hexane: DCM (24:10:50:15) as an eluent. From the reaction mixture, insoluble DCU was filtered, and the filtrate was separated. The solid product was obtained by evaporating the solvent in a rotary evaporator and further purification was carried out by flash chromatography using acetonitrile/MeOH (25:1) as an eluent. The same synthetic procedure was adopted to prepare the conjugates of flurbiprofen–sulfa drugs.

#### 3.1.3. Analytical Details of Ibuprofen–Sulfa Drug Conjugates 

##### 2-(4-Isobutylphenyl)-*N*-(4-sulfamoylphenyl)propanamide (**4**) [29] 

White crystalline solid; yield (%): 78.8; m.p. (°C): 147–150; R*_f_*:0.73; IR (ATR, υ cm^−1^): 3463 (sulfonyl-NH), 3011 (aromatic, =C-H), 2921 (amide-NH), 1710 (-C=O), 1594 (-CH=CH-), 1366 (asymmetric, -NH-S=O), 1141 (symmetric, -NH-S=O), 1027 (-S=O); ^1^H NMR (400 MHz, DMSO-*d*_6_): δH 10.48 (brs, 1H, NH), 7.55–7.38 (m, *J* = 8.0 Hz, 6H, ArH), 6.90 (brs, 2H, NH_2_), 6.58 (d, *J* = 8.0 Hz, 2H, ArH), 3.94 (q, *J* = 4.0 Hz, 1H, CH), 2.50 (obscured by DMSO signal, CH_2_), 1.88–1.83 (m, 1H, CH), 1.47 (d, *J* = 8.0 Hz, 3H, CH_3_), 1.41 (d, *J* = 8.0 Hz, 6H, (CH_3_)_2_); ^13^C NMR (100 MHz, DMSO- *d*_6_): δC 172.6, 143.9, 142.4, 138.9, 133.7, 129.2, 128.3, 127.2, 119.3, 50.2, 46.0, 25.9, 24.9, 18.9. Anal. Calculated for C_19_H_24_N_2_O_3_S (360.47 g/mol): C, 63.31; H, 6.71; N, 7.77; O, 13.32; S, 8.99% found; C, 63.55; H, 6.77; N, 7.97; O, 13.53; S, 9.01%.

##### N-(4-(N-(3,4-Dimethylisoxazol-5-yl)sulfamoyl)phenyl)-2-(4-isobutylphenyl)propanamide (**5**) 

White crystalline solid; yield (%): 76.1; m.p. (°C): 114–116; R*_f_*:0.71; IR (ATR, υ cm^−1^): 3465 (sulfonyl-NH), 3016 (aromatic, =C-H), 2926 (amide-NH), 2836 (O-CH_3_), 1710 (-C=O), 1628 (imine –C=N-), 1594 (-CH=CH-), 1361 (asymmetric, -NH-S=O), 1138 (symmetric, -NH-S=O), 1024 (-S=O); ^1^H NMR (400 MHz, DMSO-*d*_6_): δH 10.48 (brs, 1H, NH), 7.55–7.38 (m, *J* = 8.0 Hz, 6H, ArH), 6.58 (d, *J* = 8.0 Hz, 2H, ArH), 5.81 (brs, 1H, NH), 3.94 (q, *J* = 4.0 Hz, 1H, CH), 2.50 (obscured by DMSO signal, CH_2_), 2.08 (s, 6H, (CH_3_)_2_), 1.83–1.71 (m, 1H, CH), 1.62 (d, *J* = 8.0 Hz, 3H, CH_3_), 1.42 (d, *J* = 8.0 Hz, 6H, (CH_3_)_2_); ^13^C NMR (100 MHz, DMSO-*d*_6_): δC 177.8, 167.7, 156.8, 153.8, 143.9, 142.4, 138.9, 133.9, 129.2, 128.3, 127.2, 113.1, 53.8, 52.4, 25.9, 24.9, 18.9, 10.8, 6.3. Anal. Calculated for C_24_H_29_N_3_O_4_S (455.57 g/mol): C, 63.28; H, 6.42; N, 9.22; O, 14.05; S, 7.04% found; C, 63.51; H, 6.58; N, 9.51; O, 14.35; S, 7.15%

##### 2-(4-Isobutylphenyl)-N-(4-(N-(thiazol-2-yl)sulfamoyl)phenyl)propanamide (**6**)

White crystalline solid; yield (%): 74.5; m.p. (°C): 184–185; R*_f_*:0.80; IR (ATR, υ cm^−1^): 3461 (sulfonyl-NH), 3011 (aromatic, =C-H), 2919 (amide-NH), 1708 (-C=O), 1622 (imine –C=N-), 1594 (-CH=CH-), 1361 (asymmetric, -NH-S=O), 1138 (symmetric, -NH-S=O), 1021 (-S=O); ^1^H NMR (400 MHz, DMSO-*d*_6_): δH 10.45 (brs, 1H, NH), 7.73–7.50 (m, 2H, ArH), 7.41 (d, 1H, *J* = 8.0 Hz, ArH), 7.38 (d, 1H, *J* = 8.0 Hz, ArH), 7.29 (d, 2H, *J* = 2.4 Hz, ArH), 7.19 (td, 2H, *J* = 8.0, 2.4 Hz, ArH), 6.76 (d, 1H, *J* = 8.0 Hz, =N-CH), 6.55 (d, 1H, *J* = 8.0 Hz, -S-CH), 5.84 (brs, 1H, NH), 3.93 (q, 1H, *J* = 8.0 Hz, CH), 2.50 (obscured by DMSO signal, CH_2_), 1.87–1.70 (m, 1H, CH), 1.58 (d, *J* = 8.0 Hz, 3H, CH_3_), 1.42 (d, *J* = 8.0 Hz, 6H, (CH_3_)_2_); ^13^C NMR (100 MHz, DMSO-*d*_6_): δC 172.6, 153.8, 143.8, 142.4, 137.5, 135.4, 131.2, 129.1, 129.1 128.2, 119.2, 112.9, 50.0, 46.0, 32.2, 24.9, 18.8; Anal. Calculated for C_22_H_25_N_3_O_3_S_2_ (443.58 g/mol): C, 59.57; H, 5.68; N, 9.47; O, 10.82; S, 14.46% found: C, 59.47; H, 5.67; N, 9.61; O, 10.95; S, 14.41%. 

##### 2-(4-Isobutylphenyl)-N-(4-(N-(pyrimidin-2-yl) sulfamoyl)phenyl)propanamide (**7**)

White crystalline solid; yield (%): 74.6; m.p. (°C): 171–173; R*_f_*:0.71; IR (ATR, υ cm^−1^): 3461 (sulfonyl-NH), 3018 (aromatic, =C-H), 2921 (amide-NH), 1708 (-C=O), 1621 (imine –CH=N-), 1588 (-CH=CH-), 1359 (asymmetric, -NH-S=O), 1139 (symmetric, -NH-S=O), 1023 (-S=O); ^1^H NMR (400 MHz, DMSO-*d*_6_): δH 8.48 (d, *J* = 4.0 Hz, 2H, ArH), 7.62 (d, *J* = 8.0 Hz, 2H, ArH), 7.55–7.44 (m, 4H, ArH), 7.19 (brs, 1H, NH), 7.01 (t, *J* = 4.0 Hz, ArH), 6.56 (d, *J* = 8.0 Hz, 2H, ArH), 6.00 (brs, 1H, NH), 3.94 (q, *J* = 4.0 Hz, CH), 2.5 (obscured by DMSO signal, CH_2_), 1.73–1.53 (m, 1H, CH), 1.42 (d, *J* = 12.0 Hz, 3H, CH_3_), 1.24 (d, *J* = 12.0 Hz, 6H, (CH_3_)_2_); ^13^C NMR (100 MHz, DMSO-*d*_6_): δC 171.2, 168.2, 158.2, 143.4, 141.3, 131.3, 129.2, 128.2, 127.7, 126.8, 124.6, 115.7, 50.2, 43.4, 25.8, 24.9, 18.9. Anal. Calculated for C_23_H_26_N_4_O_3_S (438.54 g/mol): C, 62.99; H, 5.98; N, 12.87; O, 10.95; S, 7.31% found; C, 62.90; H, 5.99; N, 12.82; O, 10.94; S, 7.32%

##### 2-(4-Isobutylphenyl)-N-(4-(N-(4-methylpyrimidin-2yl)sulfamoyl)phenyl)propanamide (**8**) 

White crystalline solid; yield (%): 73.5; m.p. (°C): 152–154; R*_f_*:0.75; IR (ATR, υ cm^−1^): 3463 (sulfonyl-NH), 3021 (aromatic, =C-H), 2924 (amide-NH), 2836 (O-CH_3_), 1708 (-C=O), 1618 (imine –C=N-), 1591 (-CH=CH-), 1362 (asymmetric, -NH-S=O), 1142 (symmetric, -NH-S=O), 1031 (-S=O); ^1^H NMR (400 MHz, DMSO-*d*_6_): δH 8.31 (d, *J* = 4.0 Hz, 1H, ArH), 7.62 (d, *J* = 8.0 Hz, 2H, ArH), 7.55–7.44 (m, 4H, ArH), 7.19 (brs, 1H, NH), 6.58 (d, *J* = 4.0 Hz, 1H, ArH), 6.55 (d, *J* = 8.0 Hz, 2H, ArH), 6.00 (brs, 1H, NH), 3.94 (q, *J* = 4.0 Hz, CH), 2.5 (obscured by DMSO signal, CH_2_), 2.31 (s, 3H, CH_3_), 1.88–1.83 (m, 1H, CH), 1.56 (d, *J* = 12.0 Hz, 3H, CH_3_), 1.41 (d, *J* = 12.0 Hz, 6H, (CH_3_)_2_); ^13^C NMR (100 MHz, DMSO-*d*_6_): δC 171.2, 168.7, 160.4, 143.4, 141.3, 131.3, 129.2, 128.2, 127.7, 126.8, 122.5, 124.6, 112.8, 110.5, 50.2, 43.4, 32.0, 25.9, 24.9, 18.9; Anal. Calculated for C_24_H_28_N_4_O_3_S (452.57 g/mol): C, 63.99; H, 6.24; N, 12.38; O, 10.61; S, 7.08% found; C, 63.44; H, 6.44; N, 12.30; O, 10.71; S, 7.25%.

##### 2-(4-Isobutylphenyl)-N-(4-(N-(5-methylisoxazol-3-yl)sulfamoyl)phenyl)propanamide (**9**) [30] 

White crystalline solid; yield (%): 71.2; m.p. (°C): 150–153; R*_f_*:0.83; IR (ATR, υ cm^−1^): 3459 (sulfonyl-NH), 3022 (aromatic, =C-H), 2923 (amide-NH), 1712 (-C=O), 1617 (imine –C=N-), 1590 (-CH=CH-), 1361 (asymmetric, -NH-S=O), 1138 (symmetric, -NH-S=O), 1031 (-S=O); ^1^H NMR (400 MHz, DMSO-*d*_6_): δH 10.49 (brs, 1H, NH), 7.58–7.44 (m, 5H, ArH), 7.36 (d, *J* = 12.0 Hz, 2H, ArH), 6.60 (d, *J* = 12.0 Hz, 2H, ArH), 6.11 (brs, 1H, NH), 3.94 (q, *J* = 4.0 Hz, CH), 2.5 (obscured by DMSO signal, CH_2_), 2.08 (s, 3H, CH_3_), 1.88–1.83 (m, 1H, CH), 1.56 (d, *J* = 12.0 Hz, 3H, CH_3_), 1.41 (d, *J* = 8.0 Hz, 6H, (CH_3_)_2_); ^13^C NMR (100 MHz, DMSO-*d*_6_): δC 170.3, 160.4, 158.5, 143.9, 142.4, 138.9, 133.9, 129.2, 128.3, 127.2, 115.1, 95.8, 50.2, 46.0, 32.3, 24.9, 19.7, 12.5; Anal. Calculated for C_23_H_27_N_3_O_4_S (441.55 g/mol): C, 62.56; H, 6.16; N, 9.52; O, 14.49; S, 7.26% found; C, 62.91; H, 6.32; N, 9.71; O, 14.74; S, 7.20%

##### N-(4-(N-Acetylsulfamoyl)phenyl)-2-(4-isobutylphenyl)propanamide (**10**)

White crystalline solid; yield (%): 79.4; m.p. (°C): 152–155; R*_f_*:0.75; IR (ATR, υ cm^−1^): 3458 (sulfonyl-NH), 3021 (aromatic, =C-H), 2921 (amide-NH), 1710 (-C=O), 1594 (-CH=CH-), 1361 (asymmetric, -NH-S=O), 1142 (symmetric, -NH-S=O), 1031 (-S=O); ^1^H NMR (500 MHz, DMSO-*d*_6_): ^1^H NMR (400 MHz, DMSO-*d*_6_) δH 8.11 (brs, 1H, NH), 7.41 (d, *J* = 8.0 Hz, 2H, ArH), 7.18 (d, *J* = 8.0 Hz, 2H, ArH), 7.05 (d, *J* = 8.0 Hz, 2H, ArH), 6.48 (d, *J* = 8.0 Hz, 2H, ArH), 5.57 (brs, 1H, NH), 3.88 (q, *J* = 4.0 Hz, CH), 2.5 (obscured by DMSO signal, CH_2_), 1.88–1.83 (m, 1H, CH), 1.73 (s, 3H, CH_3_), 1.35 (d, *J* = 8.0 Hz, 6H, (CH_3_)_2_).^13^C NMR (100 MHz, DMSO-*d*_6_): δC 172.7, 168.7, 143.9, 142.4, 138.9, 133.9, 129.9, 128.3, 127.2, 119.3, 50.2, 46.0, 32.5, 25.9, 24.7, 18.9. Anal. Calculated for C_21_H_26_N_2_O_4_S (402.51 g/mol): C, 62.68; H, 6.51; N, 6.96; O, 15.90; S, 7.97% found; C, 62.88; H, 6.76; N, 6.84; O, 15.84; S, 7.95%

##### N-(4-(N-Carbamimidoylsulfamoyl)phenyl)-2-(4-isobutylphenyl)propanamide (**11**)

White crystalline solid; yield (%): 72.4; m.p. (°C): 138–139; R*_f_*:0.78; IR (ATR, υ cm^−1^): 3461 (sulfonyl-NH), 3019 (aromatic, =C-H), 2921 (amide-NH), 1710 (-C=O), 1619 (imine –CH=N-), 1594 (-CH=CH-), 1366 (asymmetric, -NH-S=O), 1141 (symmetric, -NH-S=O), 1027 (-S=O); ^1^H NMR (400 MHz, DMSO-*d*_6_): δH 8.39 (brs, 1H, NH), 7.58–7.44 (m, 6H, ArH), 7.20 (d, *J* = 8.0 Hz, 2H, ArH), 6.56 (m, 1H, NH) 5.69 (brs, 2H, NH), 3.94 (q, *J* = 8.0 Hz, CH), 2.5 (obscured by DMSO signal, CH_2_), 1.88–1.83 (m, 1H, CH), 1.57 (d, *J* = 8.0 Hz, 3H, CH_3_), 1.41 (d, *J* = 12.0 Hz, 6H, (CH_3_)_2_). ^13^C NMR (100 MHz, DMSO-*d*_6_): δC 171.2, 160.4, 131.3, 129.2, 128.2, 127.7, 126.8, 124.6, 115.6, 112.8, 53.4, 43.4, 30.5, 24.9, 19.7. Anal. Calculated for C_20_H_26_N_4_O_3_S (402.51 g/mol): C, 59.68; H, 6.51; N, 13.92; O, 11.92; S, 7.96% found; C, 59.71; H, 6.63; N, 12.80; O, 11.80; S, 7.61%

#### 3.1.4. Analytical Details of Flurbiprofen-Sulfa Drugs Conjugates 

##### 2-(2-Fluoro-[1,1′-biphenyl]-4-yl)-N-(4-sulfamoylphenyl)propanamide (**12**) [29] 

White crystalline solid; yield (%): 74.3; m.p. (°C): 147–150; R*_f_*:0.75; IR (ATR, υ cm^−1^): 3462 (sulfonyl-NH), 3018 (aromatic, =C-H), 2921 (amide-NH), 1710 (-C=O), 1594 (-CH=CH-), 1366 (asymmetric, -NH-S=O), 1141 (symmetric, -NH-S=O), 1130–1220 (C-F), 1027 (-S=O); ^1^H NMR (400 MHz, DMSO-*d*_6_): *δ*_H_ 10.48 (brs, 1H, NH), 7.55–7.17 (m, 10H, ArH), 6.60 (d, *J* = 8.0 Hz, 2H, ArH), 5.81 (brs, 2H, NH_2_), 3.94 (q, 1H, *J* = 8.0 Hz, CH), 1.42 (d, 3H, *J* = 8.0 Hz, CH_3_). ^13^C NMR (100 MHz, DMSO-*d*_6_): δC 171.2, 160.4, 143.4, 141.3, 131.3, 129.2, 128.2, 127.7, 126.8, 124.6, 123.4, 121.3, 115.7, 112.8, 43.4, 19.7. Anal. calculated for C_21_H_19_FN_2_O_3_S (398.45 g/mol): C, 63.3; H, 4.81; N, 7.03; O, 12.05; S, 8.05% found; C, 63.49; H, 4.98; N, 7.27; O, 12.15; S, 8.21%.

##### N-(4-(N-(3,4-Dimethylisoxazol-5-yl)sulfamoyl)phenyl)-2-(2-fluoro-[1,1′-biphenyl]-4-yl)propanamide (**13**) 

White crystalline solid; yield (%): 74.5; m.p. (°C): 128–130; R*_f_*:0.84; IR (ATR, υ cm^−1^): 3460 (sulfonyl-NH), 3021 (aromatic, =C-H), 2921 (amide-NH), 1712 (-C=O), 1633 (imine –CH=N-), 1594 (-CH=CH-), 1364 (asymmetric, -NH-S=O), 1138 (symmetric, -NH-S=O), 1130–1224 (C-F), 1029 (-S=O); ^1^H NMR (400 MHz, DMSO-*d*_6_): *δ*_H_ 10.49 (brs, 1H, NH), 7.55–7.31 (m, 8H, ArH), 7.22 (app dd, 2H, *J* = 8.0, 4.0 Hz, ArH), 6.58 (d, 2H, *J* = 8.0 Hz, ArH), 5.81 (brs, 1H, NH), 3.94 (q, 1H, *J* = 8.0 Hz, CH), 2.08 (s, 6H, (CH_3_)_2_), 1.42 (d, 3H, *J* = 8.0 Hz, CH_3_). ^13^C NMR (100 MHz, DMSO-*d_6_*) *δ*_C_ 171.2, 170.4, 160.4, 156.8, 143.4, 141.3, 131.3 129.2, 128.2, 127.7, 126.8, 124.6, 123.4, 121.3, 115.7, 112.8, 110.5, 43.4, 24.9, 19.7, 6.3. Anal. calculated for C_26_H_24_FN_3_O_4_S (493.55 g/mol): C, 63.27; H, 4.90; N, 8.51; O, 12.97; S, 6.5% found; C, 63.59; H, 4.95; N, 8.67; O, 12.81; S, 6.7% 

##### 2-(2-Fluoro-[1,1′-biphenyl]-4-yl)-N-(4-(N-(thiazol-2-yl)sulfamoyl)phenyl)propanamide (**14**) 

White crystalline solid; yield (%): 71.4; m.p. (°C): 137–140; R*_f_*:0.78; IR (ATR, υ cm^−1^): 3461 (sulfonyl-NH), 3018 (aromatic, =C-H), 2921 (amide-NH), 2838 (O-CH_3_), 1710 (-C=O), 1631 (imine –C=N-), 1594 (-CH=CH-), 1366 (asymmetric, -NH-S=O), 1141 (symmetric, -NH-S=O), 1130–1220 (C-F), 1027 (-S=O); ^1^H NMR (400 MHz, DMSO-*d*_6_): *δ*_H_ 10.47 (brs, 1H, NH), 8.37 (d, 1H, *J* = 8.0 Hz, ArH), 7.55–7.20 (m, 8H, ArH), 6.80 (d, 2H, *J* = 4.0 Hz, ArH), 6.58 (d, 2H, *J* = 8.0 Hz, ArH), 6.56 (d, 1H, *J* = 8.0 Hz, ArH), 3.94 (q, 1H, *J* = 8.0 Hz, CH), 1.42 (d, 3H, *J* = 8.0 Hz, CH_3_). ^13^C NMR (100 MHz, DMSO-*d_6_*): *δ*_C_ 171.2, 158.0, 153.8, 143.8, 143.7, 130.8, 129.2, 129.1, 129.1, 129.0, 128.2, 127.4, 124.5, 119.2, 115.7, 115.4, 112.9, 43.4, 18.3. Anal. calculated for C_24_H_20_FN_3_O_3_S_2_ (481.56 g/mol): C, 59.86; H, 4.19; N, 8.73; O, 9.97; S, 13.32% found; C, 60.09; H, 4.29; N, 8.87; O, 9.95; S, 13.31%. 

##### 2-(2-Fluoro-[1,1′-biphenyl]-4-yl)-N-(4-(N-(pyrimidin-2-yl)sulfamoyl)phenyl)propanamide (**15**)

White crystalline solid; yield (%): 73.2; m.p. (°C): 155–158; R*_f_*:0.73; IR (ATR, υ cm^−1^): 3461 (sulfonyl-NH), 3018 (aromatic, =C-H), 2921 (amide-NH), 1710 (-C=O), 1632 (imine –CH=N-), 1594 (-CH=CH-), 1366 (asymmetric, -NH-S=O), 1141 (symmetric, -NH-S=O), 1130–1220 (C-F), 1027 (-S=O); ^1^H NMR (400 MHz, DMSO-*d*_6_): *δ*_H_ 11.1 (brs, 1H, NH), 8.31 (d, 1H, *J* = 8.0 Hz, ArH), 7.62 (d, 2H, *J* = 8.0 Hz, ArH), 7.55–7.28 (m, 8H, ArH), 6.88 (d, 1H, *J* = 4.0 Hz, ArH), 6.55 (d, 2H, *J* = 8.0 Hz, ArH), 5.99 (brs, 1H, NH), 3.94 (q, 1H, *J* = 8.0 Hz, CH), 1.42 (d, 3H, *J* = 8.0 Hz, CH_3_). ^13^C NMR (100 MHz, DMSO-*d*_6_): δC 171.2, 168.2, 160.4, 158.2, 153.8, 143.4, 141.3, 131.3, 129.2, 128.2, 127.7, 126.8, 124.6, 123.4, 121.3, 115.7, 112.8, 110.5, 43.4, 14.9. Anal. calculated for C_25_H_21_FN_4_O_3_S (476.53 g/mol): C, 62.01; H, 4.04; N, 11.76; O, 10.07; S, 6.73% found; C, 62.29; H, 4.28; N, 11.87; O, 10.25; S, 6.51%.

##### 2-(2-Fluoro-[1,1′-biphenyl]-4-yl)-N-(4-(N-(4-methylpyrimidin-2-yl)sulfamoyl)phenyl)propanamide (**16**) 

White crystalline solid; yield (%): 68.9; m.p. (°C): 222–225; R*_f_*:0.85; IR (ATR, υ cm^−1^): 3462 (sulfonyl-NH), 3021 (aromatic, =C-H), 2923 (amide-NH), 1710 (-C=O), 1628 (imine –C=N-), 1594 (-CH=CH-), 1366 (asymmetric, -NH-S=O), 1141 (symmetric, -NH-S=O), 1130–1220 (C-F), 1027 (-S=O); ^1^H NMR (400 MHz, DMSO-*d*_6_): *δ*_H_ 11.1 (brs, 1H, NH), 8.31 (d, 1H, *J* = 8.0 Hz, ArH), 7.62 (d, 2H, *J* = 8.0 Hz, ArH), 7.55–7.28 (m, 8H, ArH), 6.88 (d, 1H, *J* = 4.0 Hz, ArH), 6.55 (d, 2H, *J* = 8.0 Hz, ArH), 3.94 (q, 1H, *J* = 8.0 Hz, CH), 2.31 (s, 3H, CH_3_). 1.42 (d, 3H, *J* = 8.0 Hz, CH_3_). ^13^C NMR (100 MHz, DMSO-*d*_6_): δC 171.2, 168.2, 160.4, 158.2, 153.8, 143.4, 141.3, 131.3, 129.2, 128.2, 127.7, 126.8, 124.6, 123.4, 121.3, 115.7, 112.8, 110.5, 43.4, 23.4, 14.9. Anal. calculated for C_26_H_23_FN_4_O_3_S (490.55 g/mol): C, 63.66; H, 4.74; N, 11.42; O, 9.78; S, 6.54% found; C, 63.89; H, 4.98; N, 11.57; O, 9.95; S, 6.76%.

##### 2-(2-Fluoro-[1,1′-biphenyl]-4-yl)-N-(4-(N-(5-methylisoxazol-3-yl)sulfamoyl)phenyl)propanamide (**17**) 

White crystalline solid; yield (%): 72.6; m.p. (°C): 125–127; R*_f_*:0.79; IR (ATR, υ cm^−1^): 3461 (sulfonyl-NH), 3018 (aromatic, =C-H), 2921 (amide-NH), 1710 (-C=O), 1634 (imine –C=N-), 1594 (-CH=CH-), 1366 (asymmetric, -NH-S=O), 1141 (symmetric, -NH-S=O), 1130–1220 (C-F), 1028 (-S=O); ^1^H NMR (400 MHz, DMSO-*d*_6_): *δ*_H_ 10.91 (brs, 1H, NH), 7.55–7.19 (m, 8H, ArH), 6.57 (d, 2H, *J* = 8.0 Hz, ArH), 6.58 (d, 2H, *J* = 8.0 Hz, ArH), 6.56 (d, 1H, *J* = 8.0 Hz, CH=CH), 6.08 (brs, 1H, NH), 3.94 (q, 1H, *J* = 4.0 Hz, CH), 1.42 (d, 3H, *J* = 8.0 Hz, CH_3_). C^13^ NMR (100 MHz, DMSO-*d_6_*) *δ*_C_ 171.2, 166.8, 160.4, 156.8, 143.8, 141.3, 131.3, 129.2, 128.2, 127.7, 126.8, 124.6, 123.4, 121.3, 115.7, 112.8, 110.5, 43.4, 24.9, 19.7. Anal. calculated for C_25_H_22_FN_3_O_4_S (479.53 g/mol): C, 62.62; H, 4.62; N, 8.76; O, 13.35; S, 6.69% found; C, 62.52; H, 4.69; N, 8.66; O, 13.25; S, 6.76%.

##### N-(4-(N-Acetylsulfamoyl)phenyl)-2-(2-fluoro-[1,1′-biphenyl]-4-yl)propanamide (**18**) 

White crystalline solid; yield (%): 72.7; m.p. (°C): 187–190; R*_f_*:0.72; IR (ATR, υ cm^−1^): 3461 (sulfonyl-NH), 3018 (aromatic, =C-H), 2921 (amide-NH), 1710 (-C=O), 1594 (-CH=CH-), 1366 (asymmetric, -NH-S=O), 1141 (symmetric, -NH-S=O), 1130–1220 (C-F), 1027 (-S=O); ^1^H NMR (400 MHz, DMSO-*d*_6_): *δ*_H_ 10.06 (brs, 1H, NH), 7.55–7.37 (m, 8H, ArH), 7.22 (d, 2H, *J* = 8.0 Hz, ArH), 6.58 (d, 2H, *J* = 8.0 Hz, ArH), 3.63 (obscured by DMSO signal), 2.08 (s, 3H, CH_3_). 1.36 (d, 3H, *J* = 8.0 Hz, CH_3_). ^13^C NMR (100 MHz, DMSO-*d_6_*) *δ*_C_ 171.2, 168.9, 160.4, 143.4, 141.3, 131.3, 129.2, 128.2, 127.7, 126.8, 124.6, 123.4, 121.3, 115.7, 112.8, 43.4, 24.9, 19.7. Anal. calculated for C_23_H_21_FN_2_O_4_S (440.49 g/mol) C, 62.72; H, 4.81; N, 6.36; F, 4.31; O, 14.53; S, 7.28% found; C, 62.61; H, 4.98; N, 6.57; F, 4.53; O, 14.75; S, 7.29%.

##### N-(4-(N-Carbamimidoylsulfamoyl)phenyl)-2-(2-fluoro-[1,1′-biphenyl]-4-yl)propanamide (**19**) 

White crystalline solid; yield (%): 68.9; m.p. (°C): 163–165; R*_f_*:0.83; IR (ATR, υ cm^−1^): 3462 (sulfonyl-NH), 3018 (aromatic, =C-H), 2923 (amide-NH), 1710 (-C=O), 1628 (imine –CH=N-), 1594 (-CH=CH-), 1366 (asymmetric, -NH-S=O), 1141 (symmetric, -NH-S=O), 1130–1220 (C-F), 1027 (-S=O); ^1^H NMR (400 MHz, DMSO-*d*_6_): *δ*_H_ 10.42 (brs, 1H, NH), 7.55–7.37 (m, 10H, ArH), 7.22 (d, 2H, *J* = 8.0 Hz, ArH), 6.69 (brs, 2H, NH_2_), 3.94 (q, 1H, *J* = 8.0 Hz, CH), 1.42 (d, 3H, *J* = 8.0 Hz, CH_3_). ^13^C NMR (100 MHz, DMSO-*d*_6_): δC 171.2, 160.4. 158.2, 143.4, 141.3, 131.3, 129.2, 128.2, 127.7, 126.8, 124.6, 123.4, 121.3, 115.7, 43.4, 15.7. Anal. calculated for C_22_H_21_FN_4_O_3_S (440.49 g/mol) C, 59.99; H, 4.81; N, 12.72; O, 10.90; S, 7.28% found; C, 59.74; H, 4.98; N, 12.87; O, 10.95; S, 7.30%.

### 3.2. Antiurease Assay

With a few minor modifications, the urease inhibition experiment was carried out as described in our past research [3,4,5]. Briefly, DMSO was used to dissolve the synthesized conjugates (inhibitors, 250–0.49 µM) and the reference urease inhibitor (thiourea). Each falcon tube contained the respective inhibitor (20 µL), buffer (K_2_HPO_4_, 100 µL, 50 mM, pH = 6.8), and jack bean urease (20 µL); each tube was mixed well; and the mixtures were incubated for 30 min at 37 °C. Each tube received 400 µL of urea (20 mM) as a substrate, which was then incubated for 10 min at the same temperature. Afterward, each tube received 400 µL of phenol reagent and 750 µL of alkali reagent containing 0.1% active chlorine and was left at 37 °C for 50 min. Following the use of a spectrophotometer (Labdex, LX210DS, London, UK) to measure absorbance of the mixture in each tube at 595 nm, Equation (1) below was used to compute the percentage of urease inhibition:

(1) $$\%\;Urease\;inhibition=\frac{1-T}{C}\times 100$$

where, T and C are the absorbance of each well containing inhibitor and blank, respectively. The results are presented as mean ± SEM. Using a regression equation where 50% inhibition was seen, the IC_50_ values of each inhibitor were determined. Each inhibitor’s binding mechanism was tested at various doses (0–20 µM) for kinetics investigations. Urea was used as a substrate in different concentrations (0.5–4.0 mM) to determine the mode of inhibition of the inhibitors, including whether they acted as uncompetitive, mixed (non-competitive), or competitive. GraphPad PRISM 7.0 was used to create Lineweaver–Burk plots, which were then used to calculate the values of Km (app), Vmax (app), and Ki (inhibition constant). 

### 3.3. Molecular Docking and Dynamics Simulation Studies

Utilizing molecular docking, the binding modes of competing inhibitors in the urease pocket were anticipated. To achieve this, the crystallographic structures of urease (PDB ID: 4H9M) was prepared for docking using the Maestro protein preparation wizard [31]. To assure structural integrity, the receptor underwent processing that included hydrogen atom addition, bond order assignment, and the establishment of a zero bond order for metals. Additionally, unnecessary protein chains and water molecules were removed. The tautomeric states of the structures were adjusted to refine the structure along with protonation at pH 7.4. The geometries of the structures were optimized by hydrogen bond assignment at a neutral pH and then minimized using the OPLS_2005 forcefield [32]. Subsequently, a site-specific grid was generated to select the co-crystal ligand. The competitive conjugates were also prepared using the LigPrep tool, and the lowest energy conformers of the conjugates were obtained for molecular docking analysis. Finally, the prepared ligands were docked to the urease receptor through the standard precision mode of Glide. The best binding poses of the conjugates complexed with urease were subjected to 50 ns using VMD [33] and NAMD [34] to explore their stability. As a starting point, the initial files required to run the simulation were prepared using the modules of Ambertools 21 [35]. The antechamber modules were used to generate the parameters of conjugates, whereas the hydrogen atoms that were missing in the protein structures were added using the Leap program [36]. After parameterization, TIP3P water molecules were added to the systems in a periodic box of 10 Å [37] and then neutralized by the addition of Na^+^ ions. The energy clashes were removed by minimizing the system using an ff14SB forcefield [38] for protein and GAFF for ligands. After minimization, the solvation was equilibrated for 10,000 steps, which was followed by temperature equilibrations at 200, 250, and 300 K. The final equilibrated systems were then subjected to a 50 ns production run, and the trajectories were stored at every 2 ps for analysis. The analysis of the MD trajectories was conducted using the BIO3D package of R [39].

## 4. Conclusions

The present work reports on the design, successfully synthesis, and characterization of 16 conjugates by coupling ibuprofen and flurbiprofen with sulfa drugs, which contain biologically important acetamide and sulfonamide scaffolds. The drug conjugates were synthesized in good yield (68.9–79.4%) from a one-step coupling reaction of ibuprofen and flurbiprofen and various substituted sulfa drugs and characterized by spectroscopic techniques. For urease, the best inhibitor was achieved for conjugate 6 (ibuprofen–sulfathiazole), with a KI value of 0.17 µM. The effective structural feature of the most active inhibitor (90.6% inhibition) comprised a thiazole-substituted sulfonamide with ibuprofen, whereas the same substituted sulfonamide with flurbiprofen showed six-fold less activity against the urease (60.4 inhibition). It is evident that the acetamide linked to phenyl-alkyl groups showed better activity than the fluoro-substituted biphenyl group. Overall, these SAR studies suggest that modifications to the aromatic ring and sulfonamide group of acetamide–sulfonamide can significantly impact its inhibitory activity against urease. Further studies are needed to explore the full potential of acetamide–sulfonamide as a urease inhibitor and to optimize its structure for maximum efficacy. Furthermore, the binding stability of the complexes was analyzed by aligning the different snapshots of the md trajectories, which indicated that the ligands remained closely bound to the protein during simulation. Additionally, ADMET prediction showed that the synthesized drug conjugates had drug-like properties, low toxicity, and adverse effects. Finally, the in-silico analysis of these conjugates (**4**–**19**) within urease-active sites rationalized the achieved biological results.

## Figures and Tables

**Table 1:** Details of urease inhibition studies by conjugates of sulfa drugs with ibuprofen and flurbiprofen.

Conjugate	IC_50_ (µM); Mean ± SEM (% Inhibition)	^a^ V_max (app)_ (µM/min)	^b^ K_m (app)_ (mM)	^c^ K_i_ (µM)	Mode of Inhibition
4	14.26 ± 0.14 (84.2)	3.64	1.66	8.20	Mixed
5	23.27 ± 0.41 (84.0)	-	-	-	-
6	9.95 ± 0.14 (90.6)	1.39	2.30	0.17	Competitive
7	50.18 ± 1.24 (71.6)	-	-	-	-
8	12.43 ± 0.54 (87.7)	3.0	0.384	3.01	Mixed
9	39.89 ± 1.17 (76.7)	-	-	-	-
10	10.27 ± 0.11 (88.9)	1.04	1.92	2.62	Mixed
11	44.46 ± 1.41 (78.7)	-	-	-	-
12	24.92 ± 0.24 (82.1)	-	-	-	-
13	35.30 ± 0.21 (84.6)	-	-	-	-
14	63.42 ± 1.15 (60.4)	-	-	-	-
15	16.74 ± 0.23 (84.1)	1.89	1.80	7.95	Competitive
16	17.48 ± 0.76 (86.1)	1.01	3.67	1.30	Mixed
17	13.39 ± 0.11 (86.1)	1.56	5.61	10.40	Competitive
18	14.78 ± 0.16 (87.1)	1.02	1.41	4.11	Mixed
19	33.83 ± 0.18 (83.8)	-	-	-	-
^d^ Thiourea	22.61 ± 0.23 (92.3)	18.61	2.18	18.18	Competitive

^a^ V_max (app)_ = intercept, the maximum rate at 20 µM of inhibitor concentration. ^b^ K_m (app)_ = slope of the line (Michaelis constant) at 20 µM of inhibitor concentration. ^c^ K_i_ (µM) = inhibition constant, ^d^ standard urease inhibitor.

**Table 2:** The physiochemical and ADMET properties of the conjugates.

Conjugate	MW	HBD	HBA	QPlogPo/w	QPlogHERG	QPPCaco	QPlogBB	QPlogKhsa
4	360.47	3	7	2.617	−5.908	237.115	−1.649	0.114
6	443.57	2	8	4.011	−6.829	568.346	−1.265	0.346
8	452.57	2	9	4.13	−7.227	510.708	−1.536	0.468
11	402.50	2	7	3.263	−6.067	196.034	−1.805	0.379
15	476.52	2	9	4.263	−8.303	445.399	−1.467	0.42
16	490.55	2	9	4.631	−8.203	522.792	−1.416	0.573
17	479.52	2	8	4.395	−7.972	342.919	−1.644	0.563
18	440.48	2	7	3.777	−7.161	203.357	−1.715	0.483

## Data Availability

This study did not report any data.

## Supplementary Materials

The following supporting information can be downloaded at: https://www.mdpi.com/article/10.3390/molecules28145389/s1, Figure S1: 2-(4-isobutylphenyl)-*N*-(4-sulfamoylphenyl)propanamide (4); Figure S2: ^1^HNMR; Figure S3: ^13^CNMR; Figure S4: N-(4-(N-(3,4-dimethylisoxazol-5-yl)sulfamoyl)phenyl)-2-(4-isobutylphenyl)propanamide (5); Figure S5: ^1^HNMR; Figure S6: ^13^CNMR; Figure S7: 2-(4-isobutylphenyl)-N-(4-(N-(thiazol-2-yl)sulfamoyl)phenyl)propanamide (6); Figure S8: ^1^HNMR; Figure S9: ^13^CNMR; Figure S10: 2-(4-isobutylphenyl)-N-(4-(N-(pyrimidin-2-yl) sulfamoyl)phenyl)propanamide (7); Figure S11: ^1^HNMR; Figure S12: ^13^CNMR; Figure S13: 2-(4-isobutylphenyl)-N-(4-(N-(4-methylpyrimidin-2yl)sulfamoyl)phenyl)propanamide (8); Figure S14: ^1^HNMR; Figure S15: ^13^CNMR; Figure S16: 2-(4-isobutylphenyl)-N-(4-(N-(5-methylisoxazol-3-yl)sulfamoyl)phenyl)propanamide (9); Figure S17: ^1^HNMR; Figure S18: ^13^CNMR; Figure S19: N-(4-(N-acetylsulfamoyl)phenyl)-2-(4-isobutylphenyl)propanamide (10); Figure S20: ^1^HNMR; Figure S21: ^13^CNMR; Figure S22: N-(4-(N-carbamimidoylsulfamoyl)phenyl)-2-(4-isobutylphenyl)propanamide (11); Figure S23: ^1^HNMR; Figure S24: ^13^CNMR; Figure S25: 2-(2-fluoro-[1,1′-biphenyl]-4-yl)-N-(4-sulfamoylphenyl)propanamide (12); Figure S26: ^1^HNMR; Figure S27: ^13^CNMR; Figure S28: N-(4-(N-(3,4-dimethylisoxazol-5-yl)sulfamoyl)phenyl)-2-(2-fluoro-[1,1′-biphenyl]-4-yl)propanamide (13); Figure S29: ^1^HNMR; Figure S30: ^13^CNMR; Figure S31: 2-(2-fluoro-[1,1′-biphenyl]-4-yl)-N-(4-(N-(thiazol-2-yl)sulfamoyl)phenyl)propanamide (14); Figure S32: ^1^HNMR; Figure S33: ^13^CNMR; Figure S34: 2-(2-fluoro-[1,1′-biphenyl]-4-yl)-N-(4-(N-(pyrimidin-2-yl)sulfamoyl)phenyl)propanamide (15); Figure S35: ^1^HNMR; Figure S36: ^13^CNMR; Figure S37: 2-(2-fluoro-[1,1′-biphenyl]-4-yl)-N-(4-(N-(4-methylpyrimidin-2-yl)sulfamoyl)phenyl)propanamide (16); Figure S38: ^1^HNMR; Figure S39: ^13^CNMR; Figure S40: 2-(2-fluoro-[1,1′-biphenyl]-4-yl)-N-(4-(N-(5-methylisoxazol-3-yl)sulfamoyl)phenyl)propanamide (17); Figure S41: ^1^HNMR; Figure S42: ^13^CNMR; Figure S43: N-(4-(N-acetylsulfamoyl)phenyl)-2-(2-fluoro-[1,1′-biphenyl]-4-yl)propanamide (18); Figure S44: ^1^HNMR; Figure S45: ^13^CNMR; Figure S46: N-(4-(N-carbamimidoylsulfamoyl)phenyl)-2-(2-fluoro-[1,1′-biphenyl]-4-yl)propanamide (19); Figure S47: ^1^HNMR; Figure S48: ^13^CNMR.
