# Supplementary material for: New Acetamide-Sulfonamide-Containing Scaffolds: Antiurease Activity Screening, Structure-Activity Relationship, Kinetics Mechanism, Molecular Docking, and MD Simulation Studies
Source: Molecules. 2023 Jul 13;28(14):5389. doi: 10.3390/molecules28145389 (PMC10386649; doi:10.3390/molecules28145389)
Supplement: Supplementary file 1 [file molecules-28-05389-s001.zip › molecules-2418217-supplementary.pdf]

Supplementary Materials

New Acetamide-Sulfonamide-Containing Scaffolds: Antiurease Activity Screening, Structure-Activity Relationship, Kinetics Mechanism, Molecular Docking, and MD Simulation Studies

Saghir Ahmad [1,2], Muhammad Abdul Qadir [1], Mahmood Ahmed [3,*], Muhammad Imran [4], Numan Yousaf [5], Tanveer A. Wani [6], Seema Zargar [7], Ijaz Ali [8] and Muhammad Muddassar [5,*]

[1] School of Chemistry, University of the Punjab, Lahore 54590, Pakistan; saghirtalib@gmail.com (S.A.); mabdulqadir@gmail.com (M.A.Q.)
[2] Department of Microbiology, Immunology and Cancer Biology, School of Medicine, University of Virginia, Charlottesville, VA 22904, USA
[3] Department of Chemistry, Division of Science and Technology, University of Education, College Road, Lahore 54770, Pakistan
[4] Kauser Abdulla Malik School of Life Sciences, Forman Christian College (A Chartered University), Lahore 54600, Pakistan; muhammadimran@fccollege.edu.pk
[5] Department of Biosciences, COMSATS University Islamabad, Park Road, Islamabad 45550, Pakistan; numanyousaf427@gmail.com
[6] Department of Pharmaceutical Chemistry, College of Pharmacy, King Saud University, P.O. Box 2457, Riyadh 11451, Saudi Arabia; twani@ksu.edu.sa
[7] Department of Biochemistry, College of Science, King Saud University, P.O. Box 222452, Riyadh 11451, Saudi Arabia; szargar@ksu.edu.sa
[8] Center for Applied Mathematics and Bioinformatics, Gulf University for Science and Technology, Mubarak Al-Abdullah 32093, Kuwait; ali.i@gust.edu.pk
* Correspondence: mahmoodresearchscholar@gmail.com (M.A.); cadd.cui@gmail.com (M.M.)

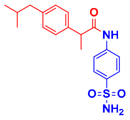

Figure S1. 2-(4-isobutylphenyl)-*N*-(4-sulfamoylphenyl)propanamide (**4**)

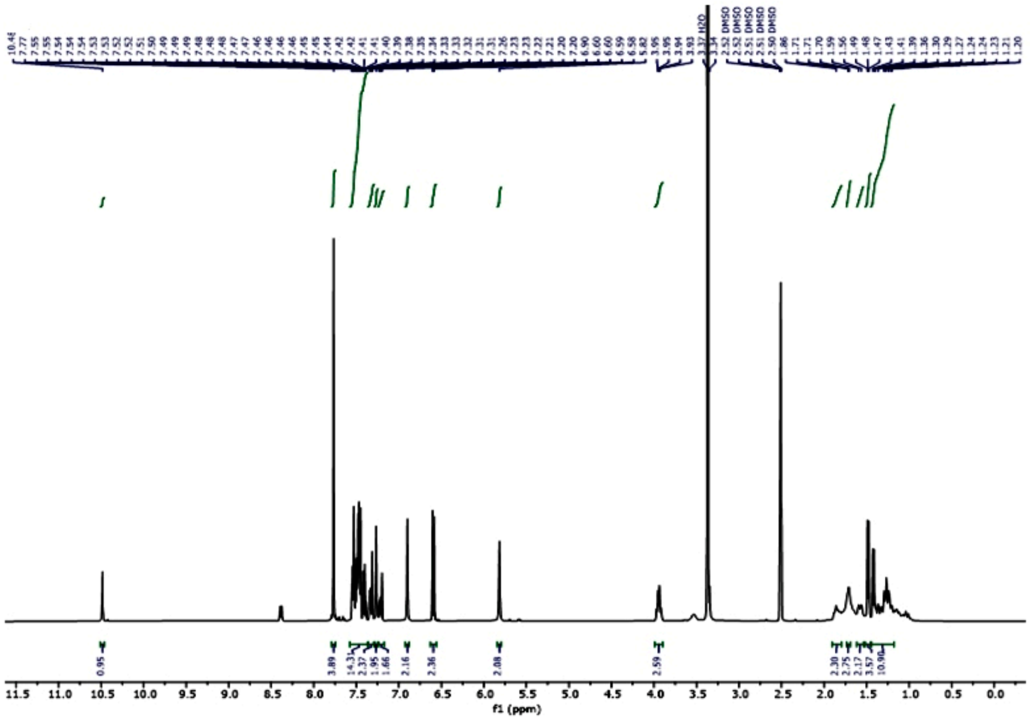

Figure S2. ^{1}HNMR

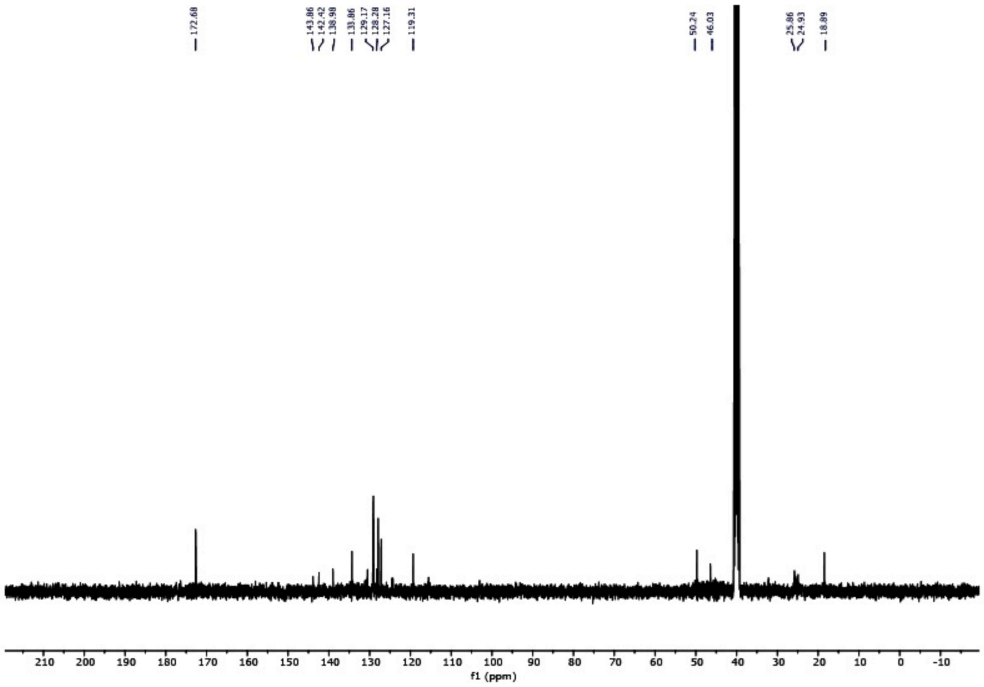

Figure S3. ^{13}CNMR

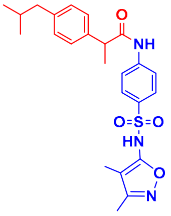

Figure S4. N-(4-(N-(3,4-dimethylisoxazol-5-yl)sulfamoyl)phenyl)-2-(4-isobutylphenyl)propanamide (5)

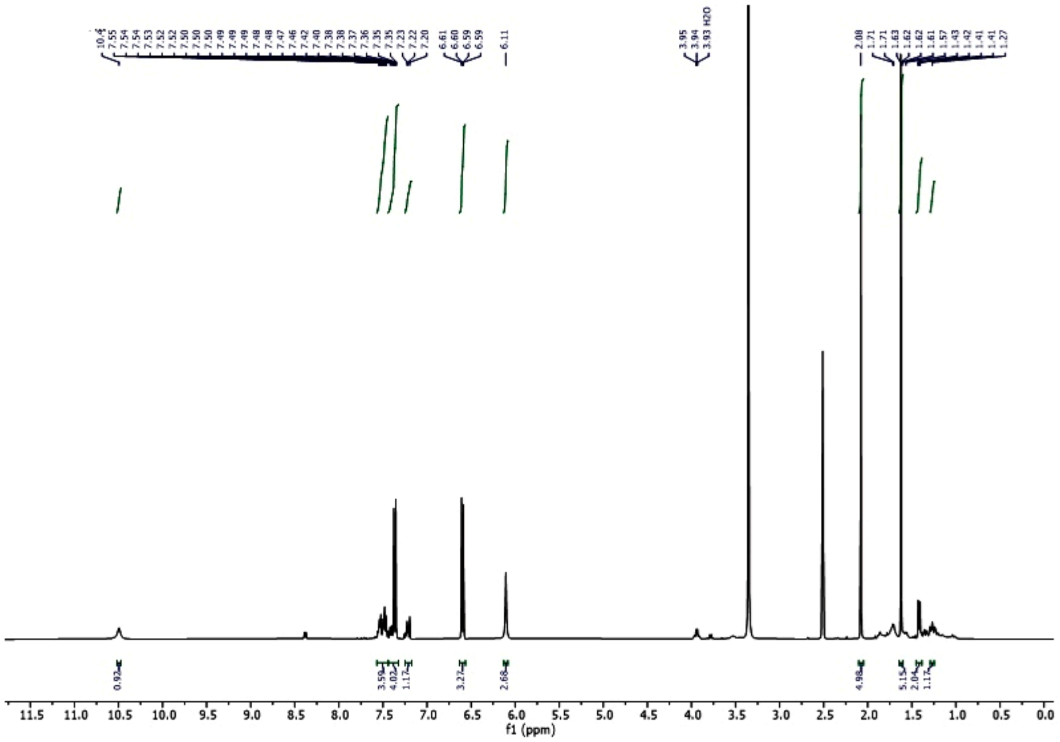

Figure S5. ^{1}HNMR

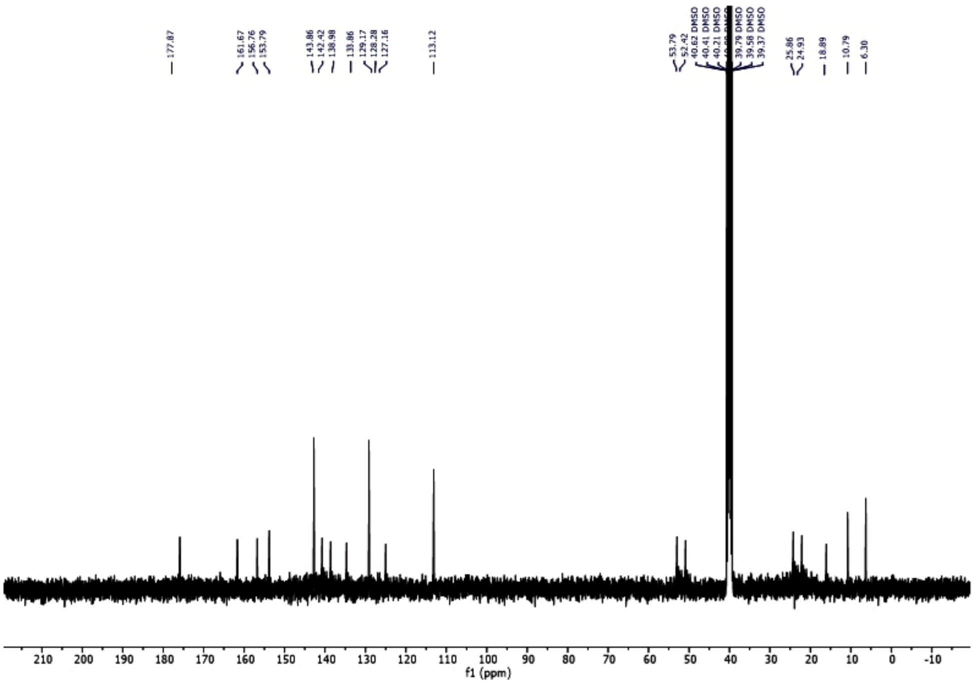

Figure S6. ^{13}CNMR

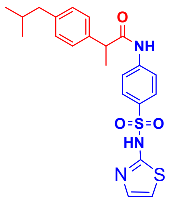

Figure S7. 2-(4-isobutylphenyl)-N-(4-(N-(thiazol-2-yl)sulfamoyl)phenyl)propanamide (**6**)

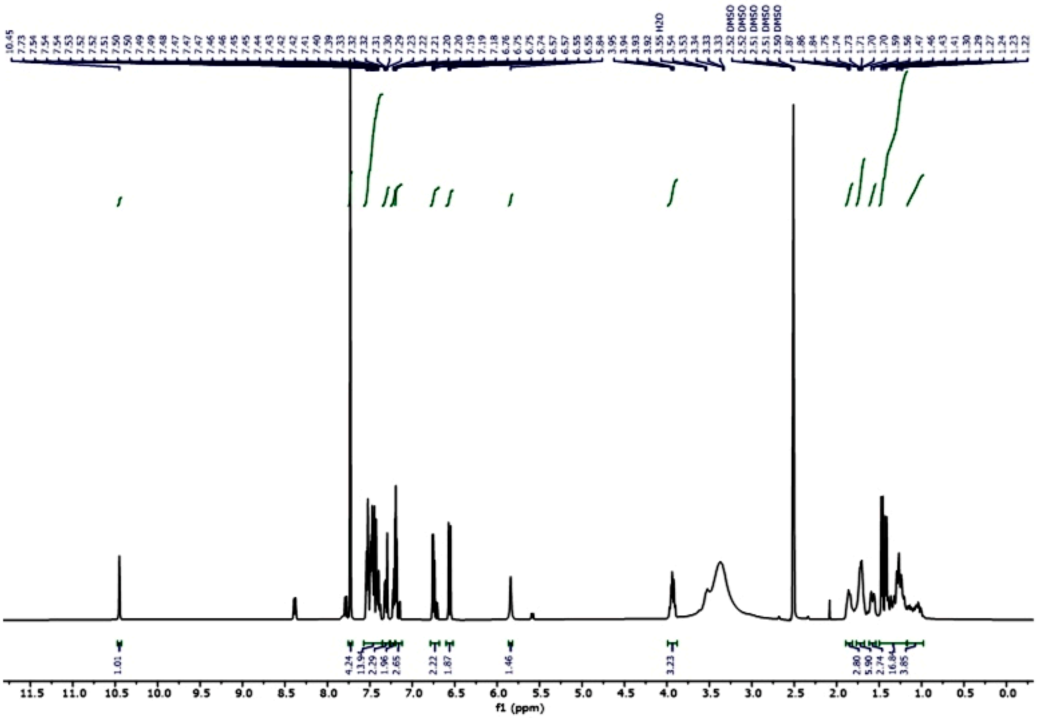

Figure S8. ^{1}HNMR

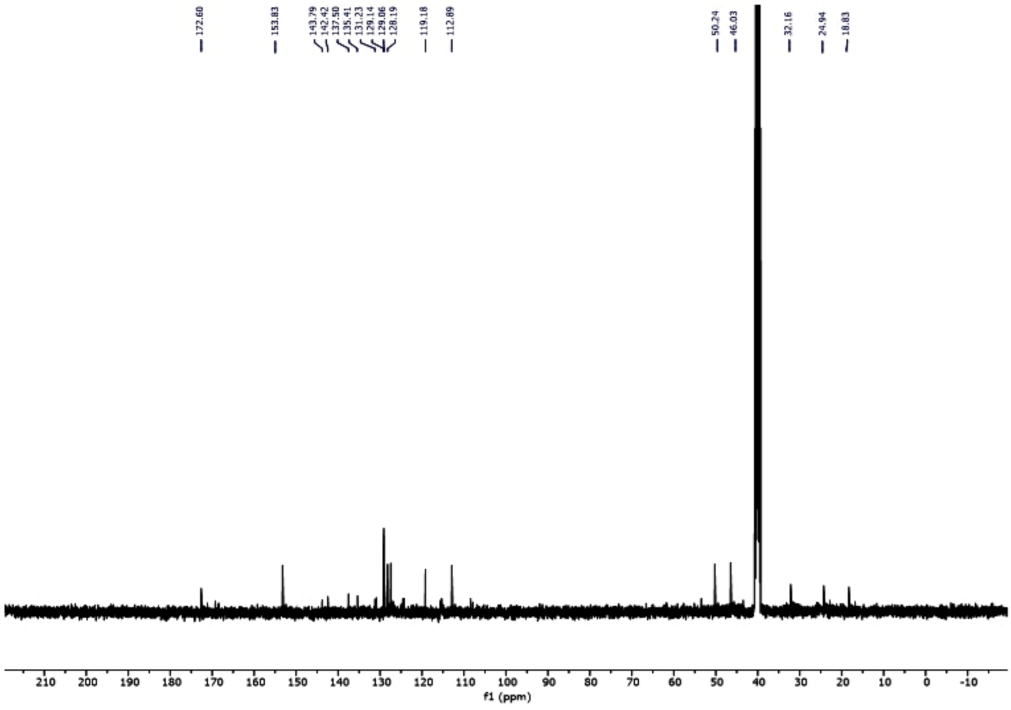

Figure S9. ^{13}CNMR

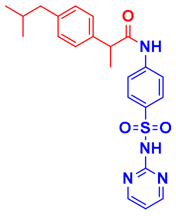

Figure S10. 2-(4-isobutylphenyl)-N-(4-(N-(pyrimidin-2-yl) sulfamoyl)phenyl)propanamide (7)

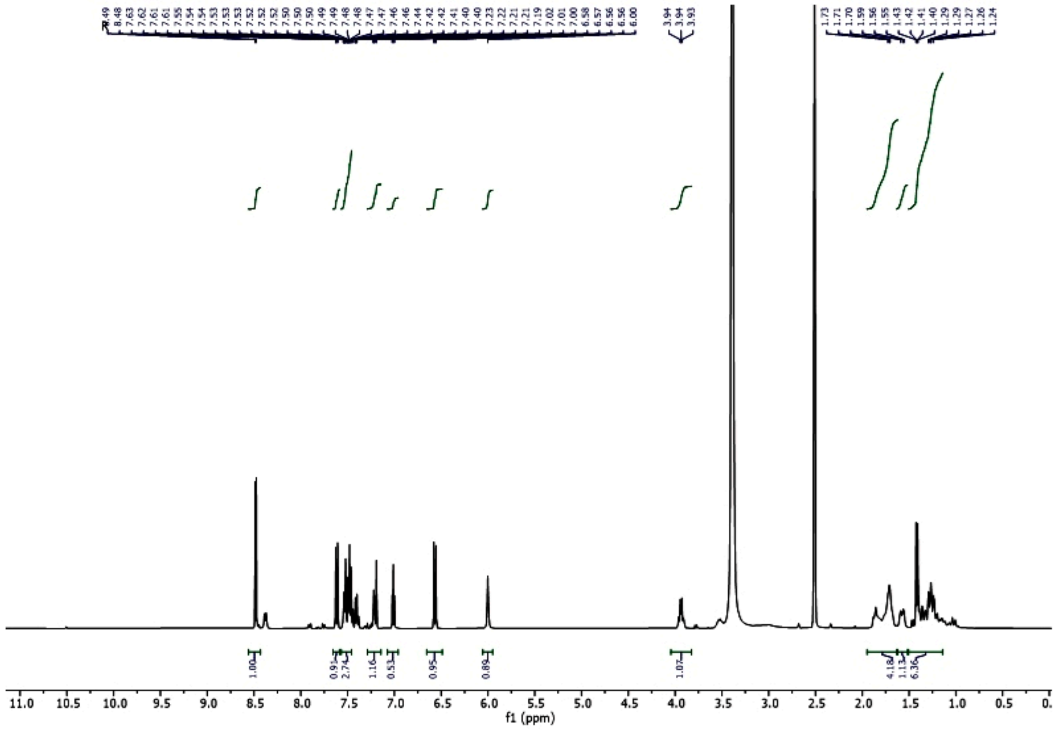

Figure S11. ^{1}HNMR

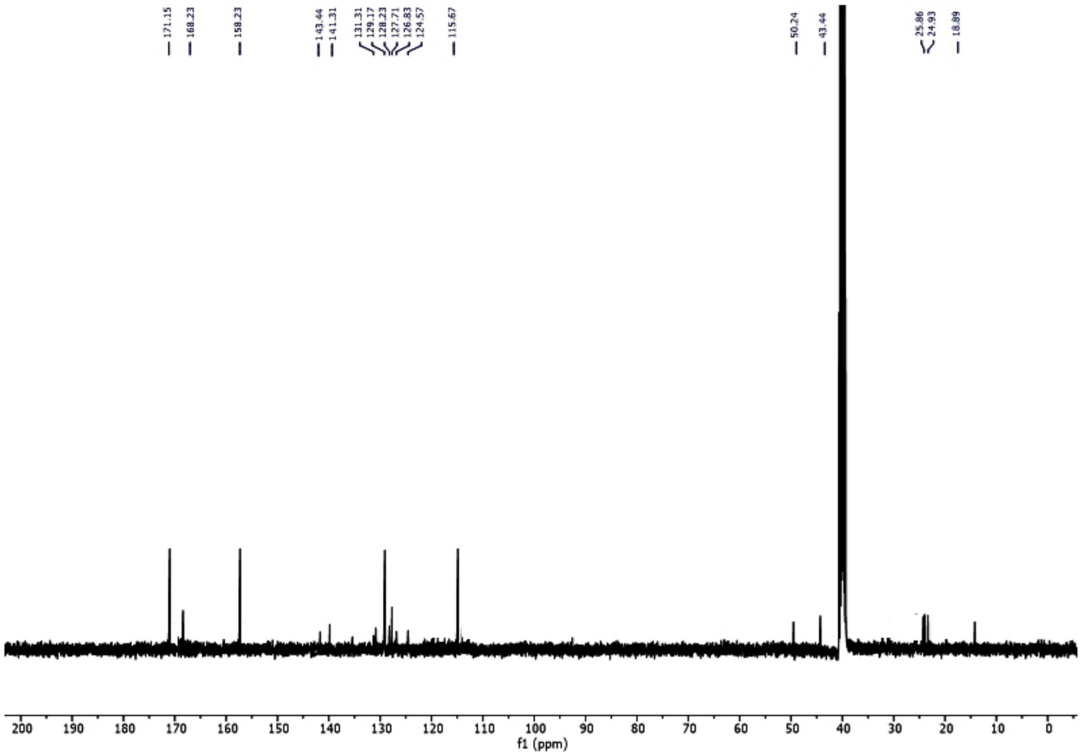

Figure S12. ^{13}CNMR

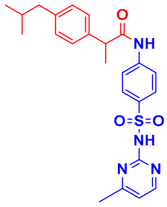

Figure S13. 2-(4-isobutylphenyl)-N-(4-(N-(4-methylpyrimidin-2yl)sulfamoyl)phenyl)propanamide (**8**)

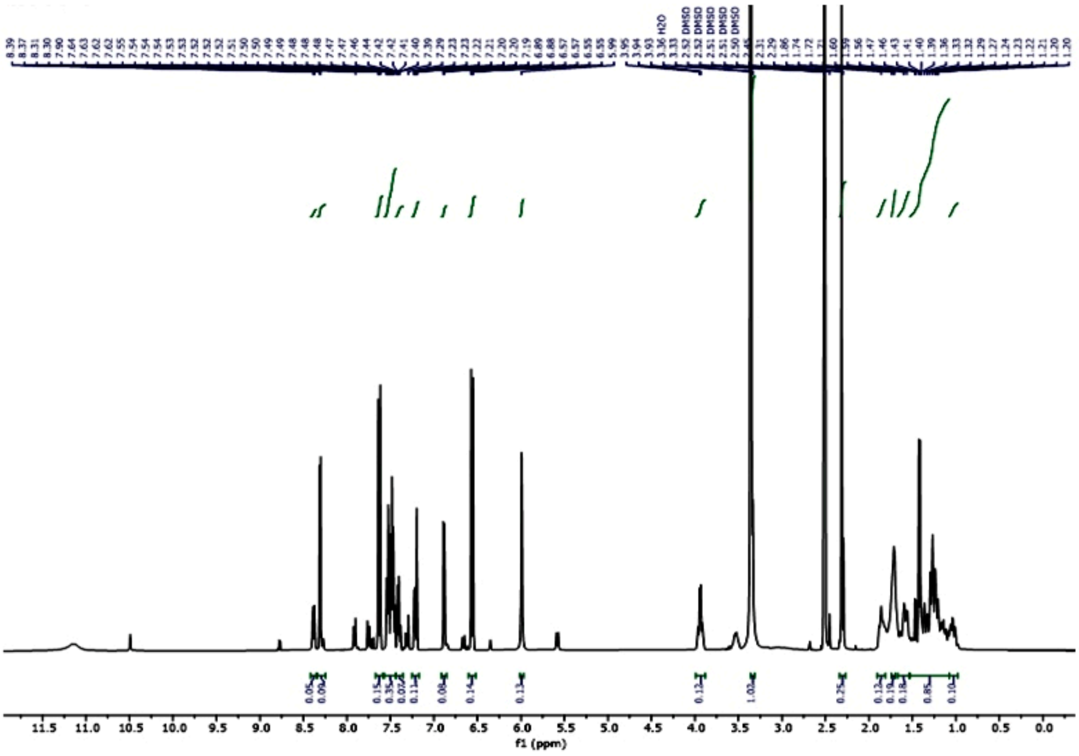

Figure S14. ^{1}HNMR

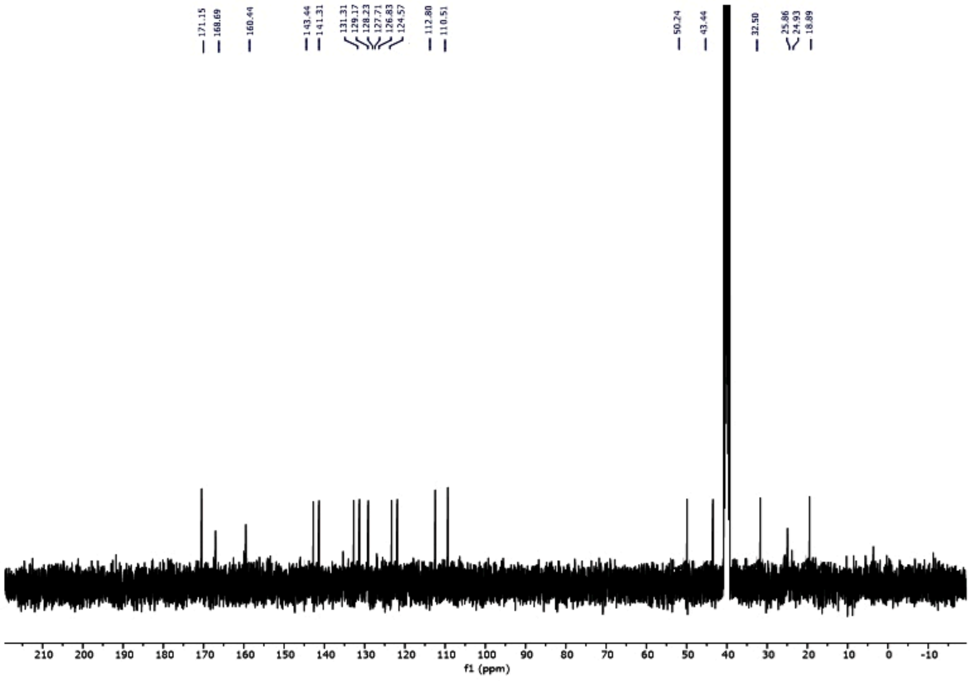

Figure S15. ^{13}CNMR

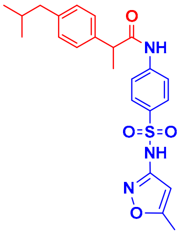

Figure S16. 2-(4-isobutylphenyl)-N-(4-(N-(5-methylisoxazol-3-yl)sulfamoyl)phenyl)propanamide (**9**)

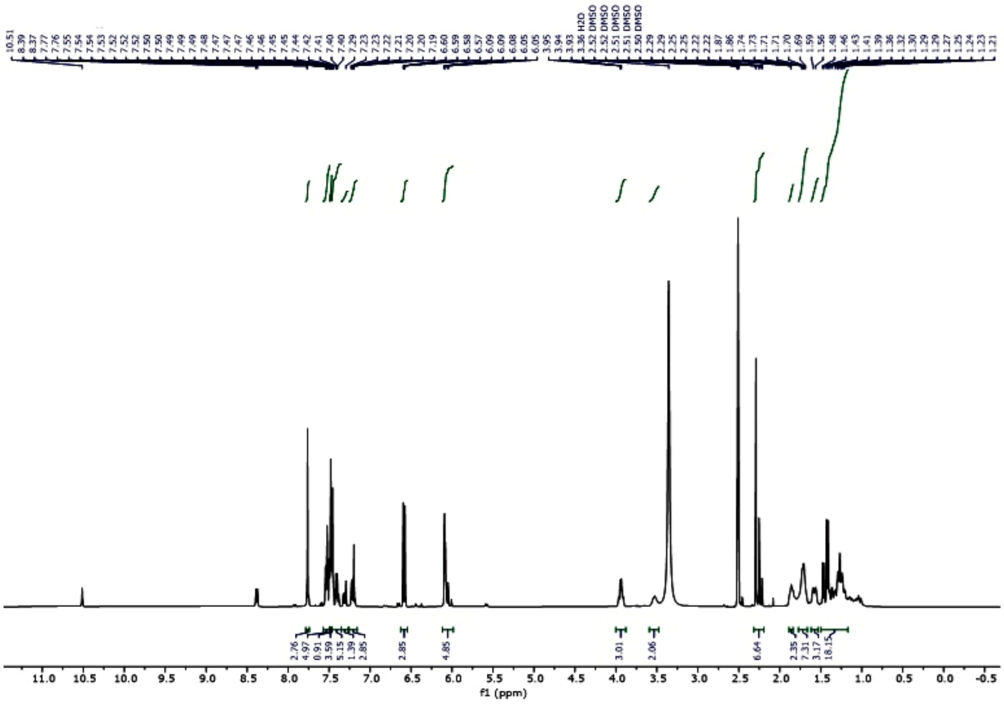

Figure S17. ^{1}HNMR

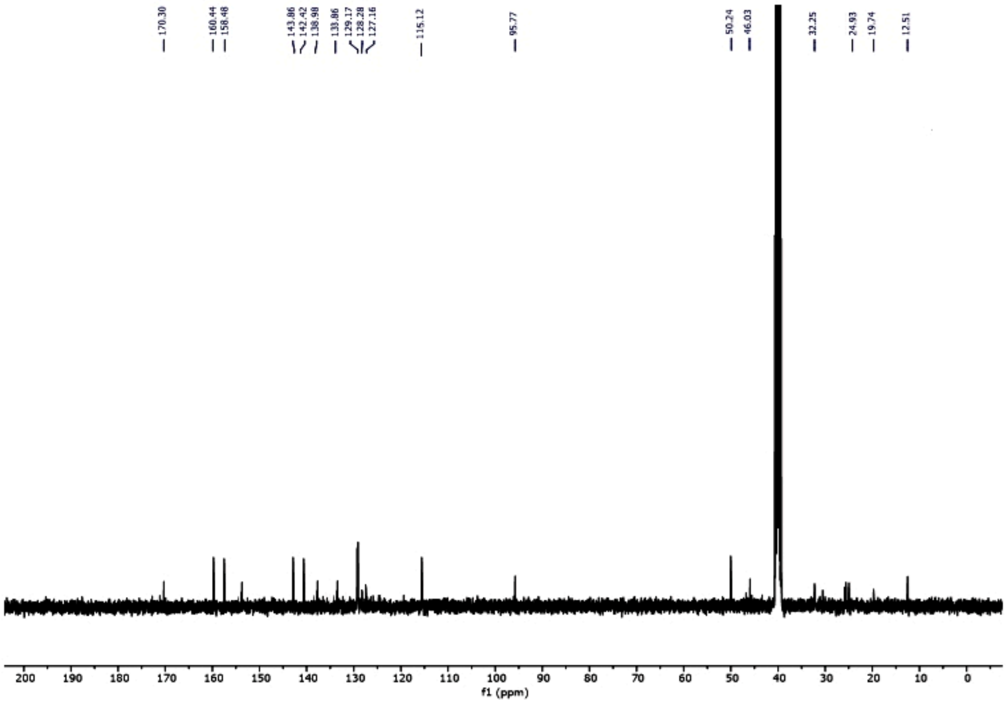

Figure S18. ^{13}CNMR

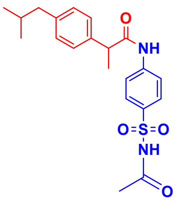

Figure S19. N-(4-(N-acetylsulfamoyl)phenyl)-2-(4-isobutylphenyl)propanamide (**10**)

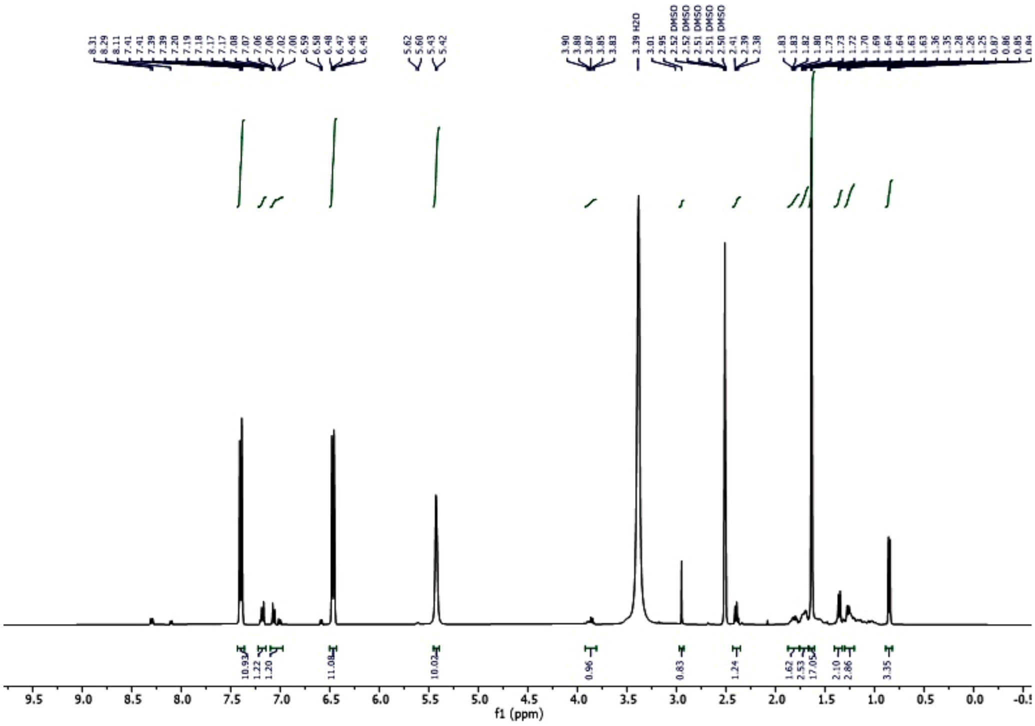

Figure S20. ^{1}HNMR

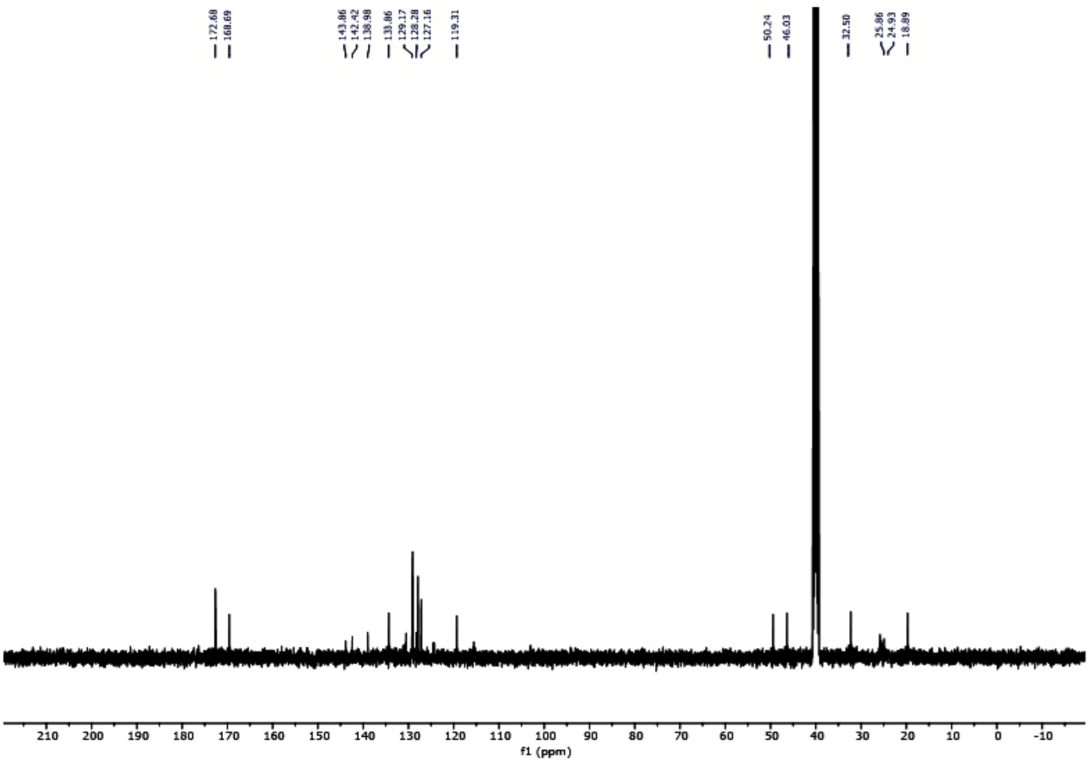

Figure S21. ^{13}CNMR

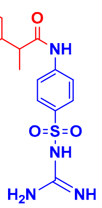

Figure S22. N-(4-(N-carbamimidoylsulfamoyl)phenyl)-2-(4-isobutylphenyl)propanamide (**11**)

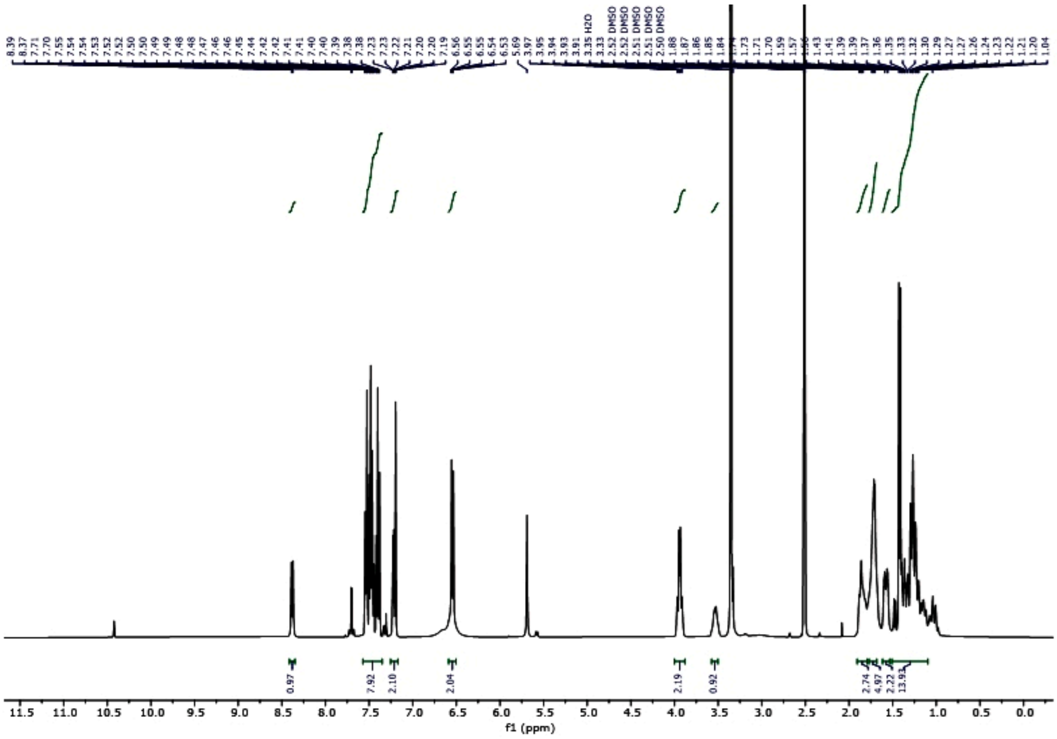

Figure S23. ^{1}HNMR

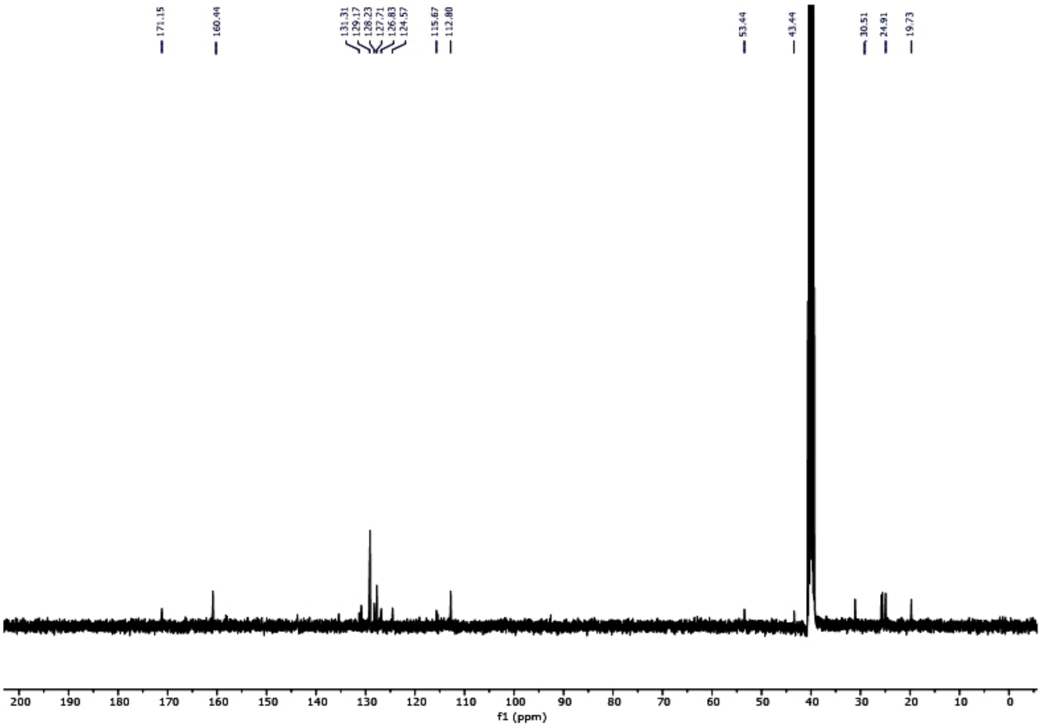

Figure S24. ^{13}CNMR

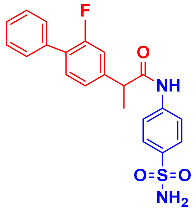

Figure S25. 2-(2-fluoro-[1,1′-biphenyl]-4-yl)-N-(4-sulfamoylphenyl)propanamide (**12**)

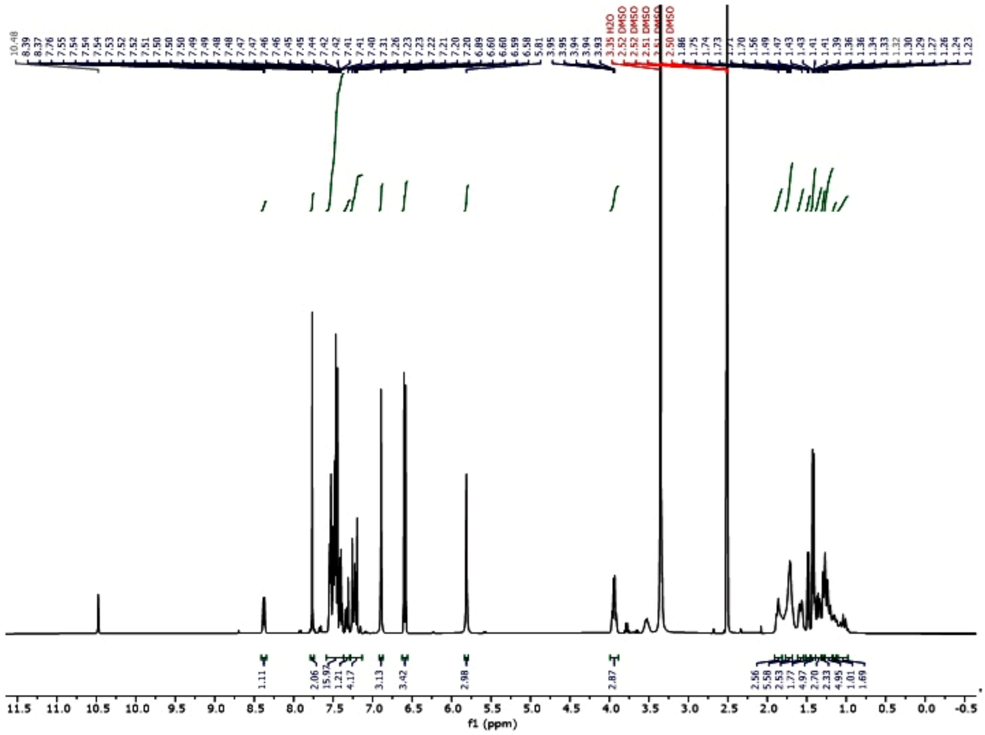

Figure S26. ^{1}HNMR

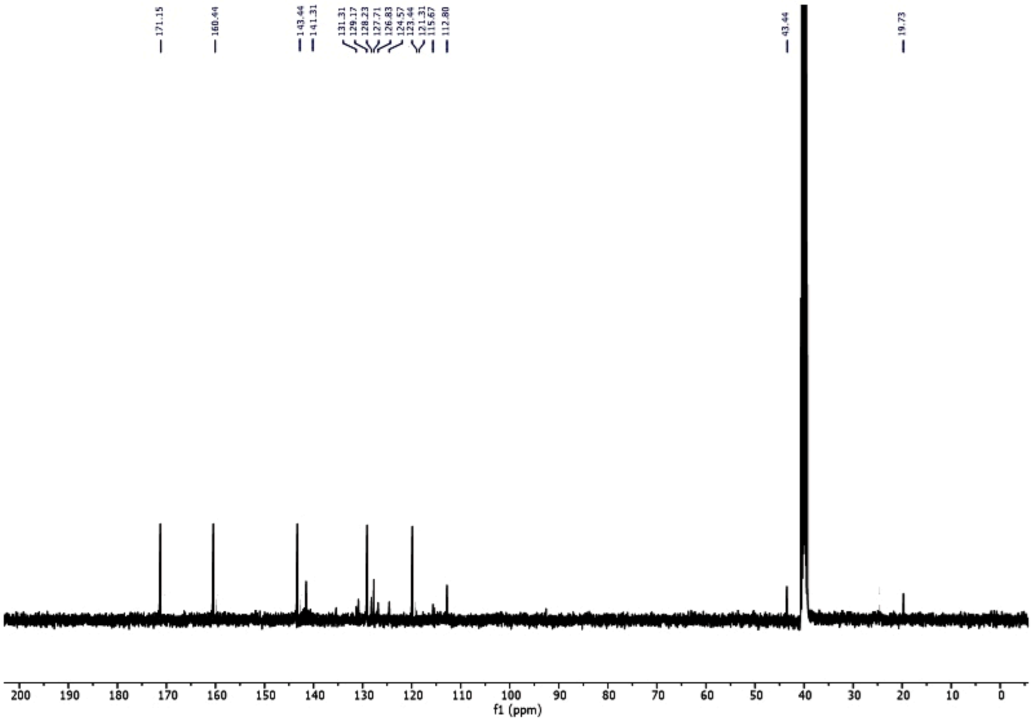

Figure S27. ^{13}CNMR

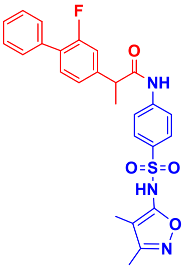

Figure S28. N-(4-(N-(3,4-dimethylisoxazol-5-yl)sulfamoyl)phenyl)-2-(2-fluoro-[1,1′-biphenyl]-4-yl)propanamide (**13**)

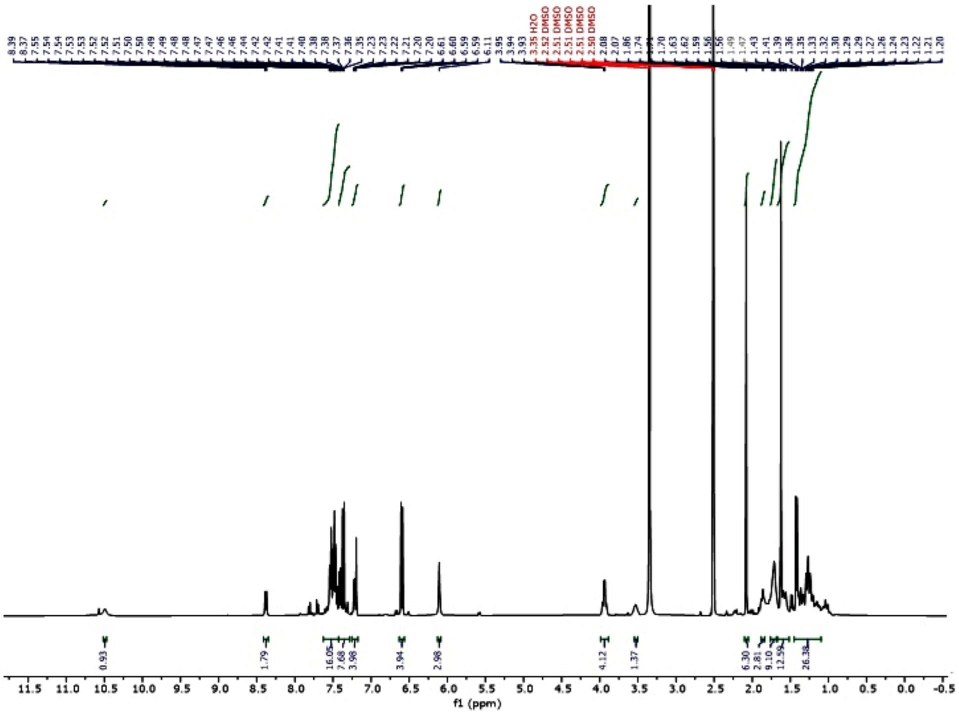

Figure S29. ^{1}HNMR

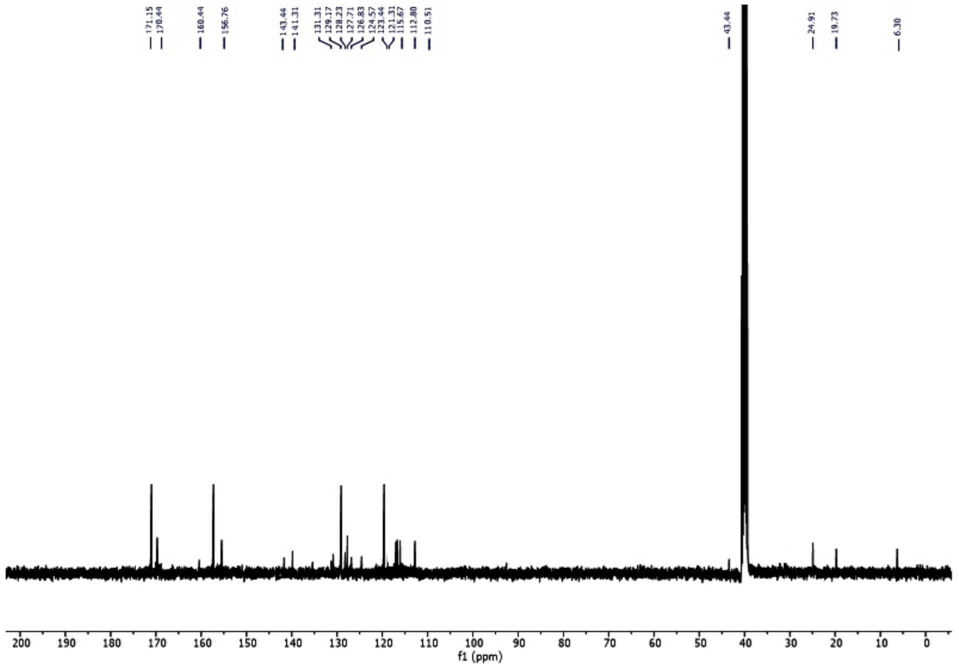

Figure S30. ^{13}CNMR

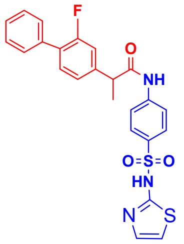

Figure S31. 2-(2-fluoro-[1,1′-biphenyl]-4-yl)-N-(4-(N-(thiazol-2-yl)sulfamoyl)phenyl)propanamide (**14**)

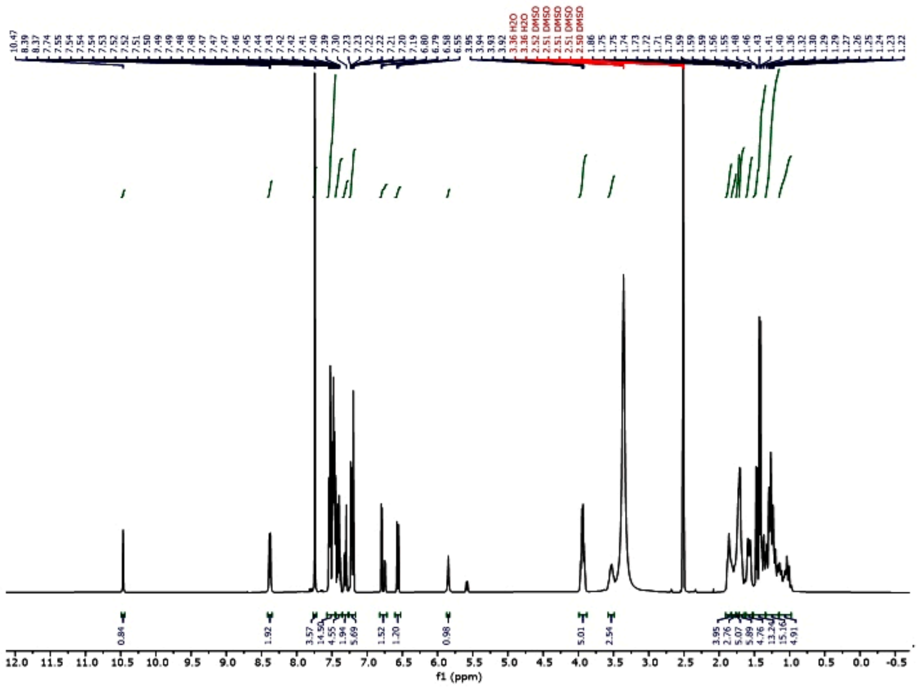

Figure S32. ^{1}HNMR

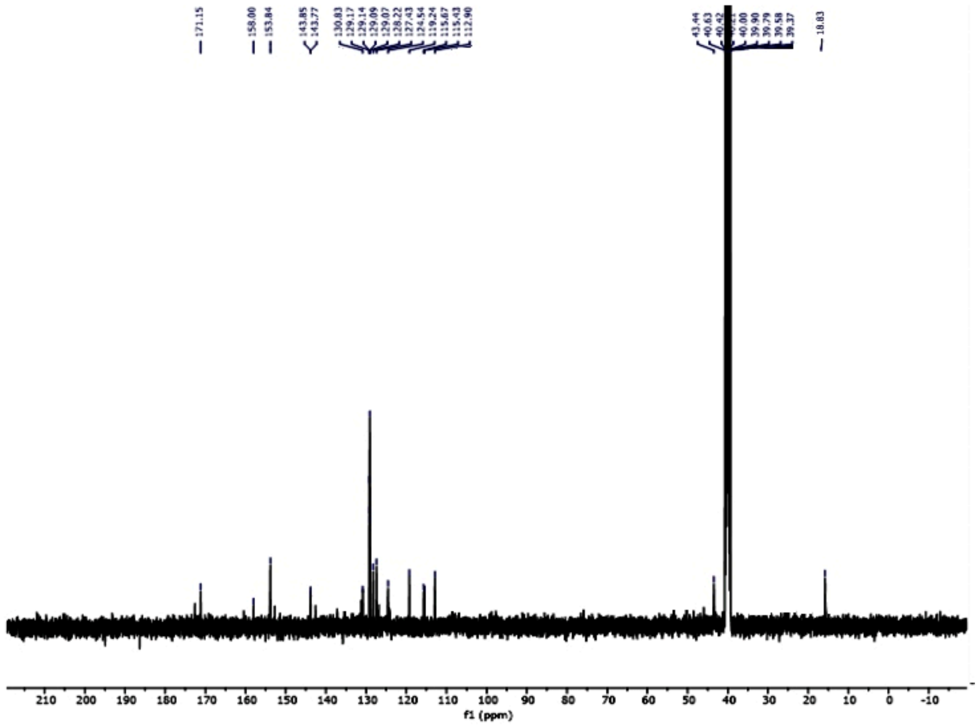

Figure S33. ^{13}CNMR

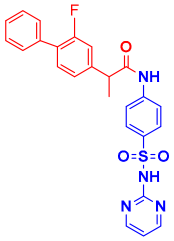

Figure S34. 2-(2-fluoro-[1,1′-biphenyl]-4-yl)-N-(4-(N-(pyrimidin-2-yl)sulfamoyl)phenyl)propanamide (**15**)

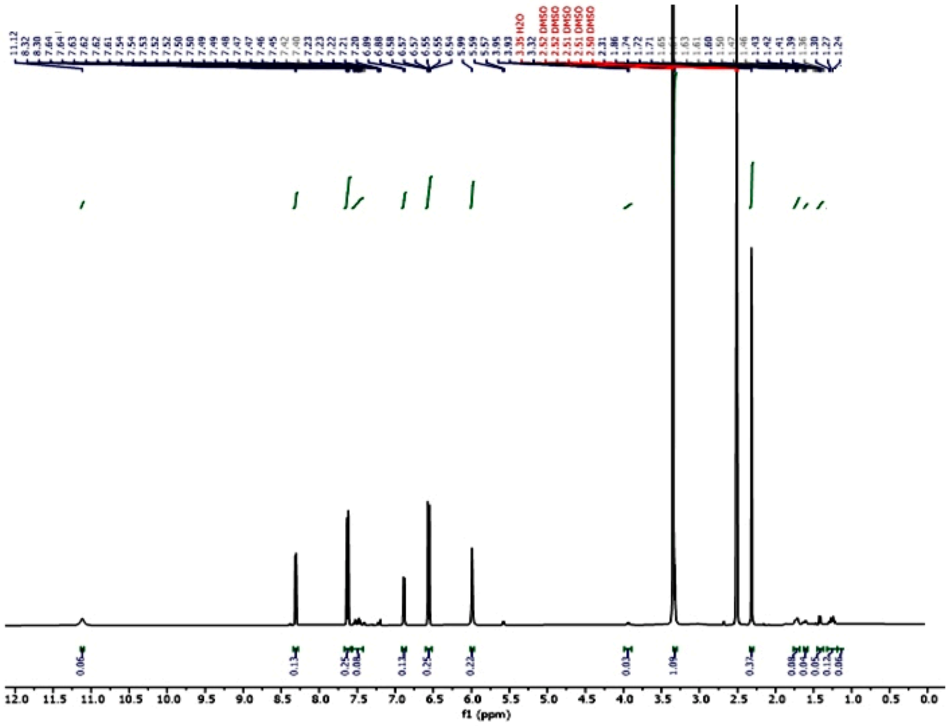

Figure S35. ^{1}HNMR

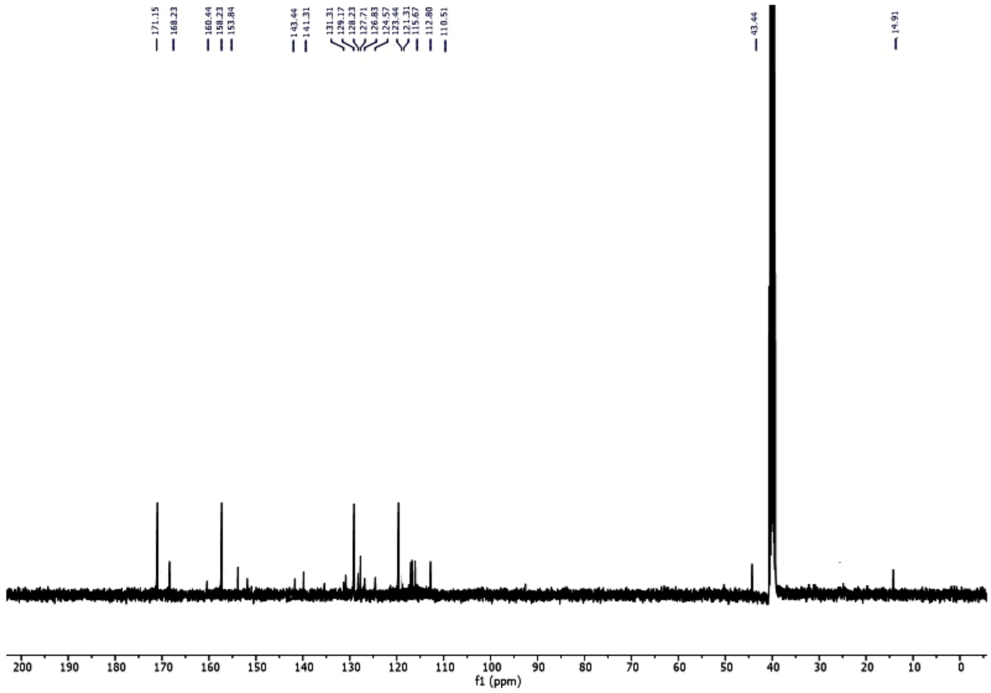

Figure S36. ^{13}CNMR

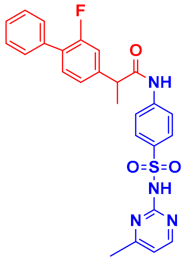

Figure S37. 2-(2-fluoro-[1,1′-biphenyl]-4-yl)-N-(4-(N-(4-methylpyrimidin-2-yl)sulfamoyl)phenyl)propanamide (**16**)

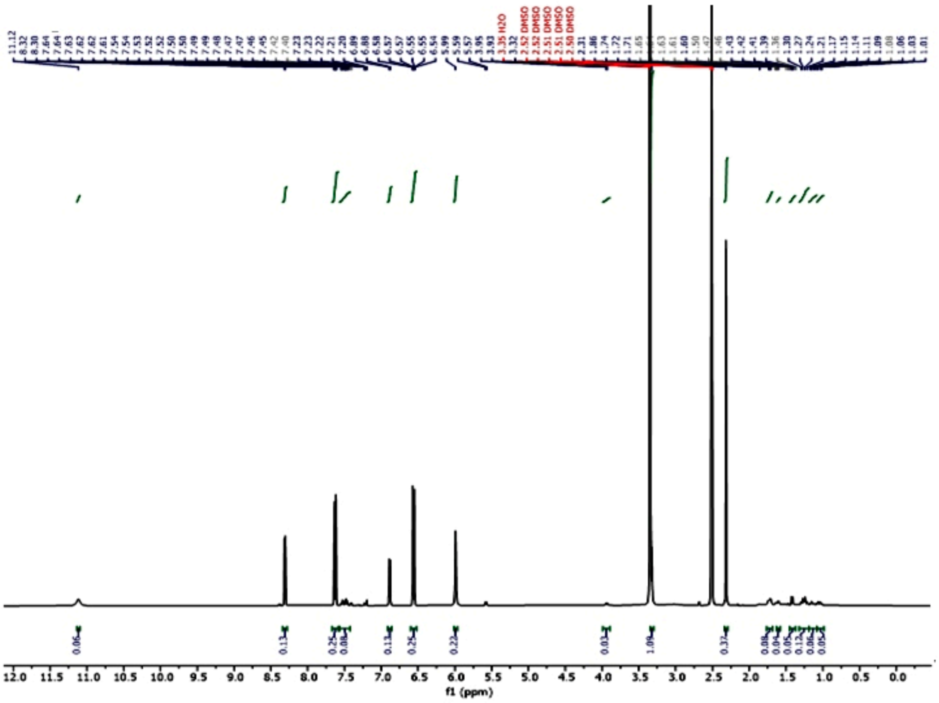

Figure S38. ^{1}HNMR

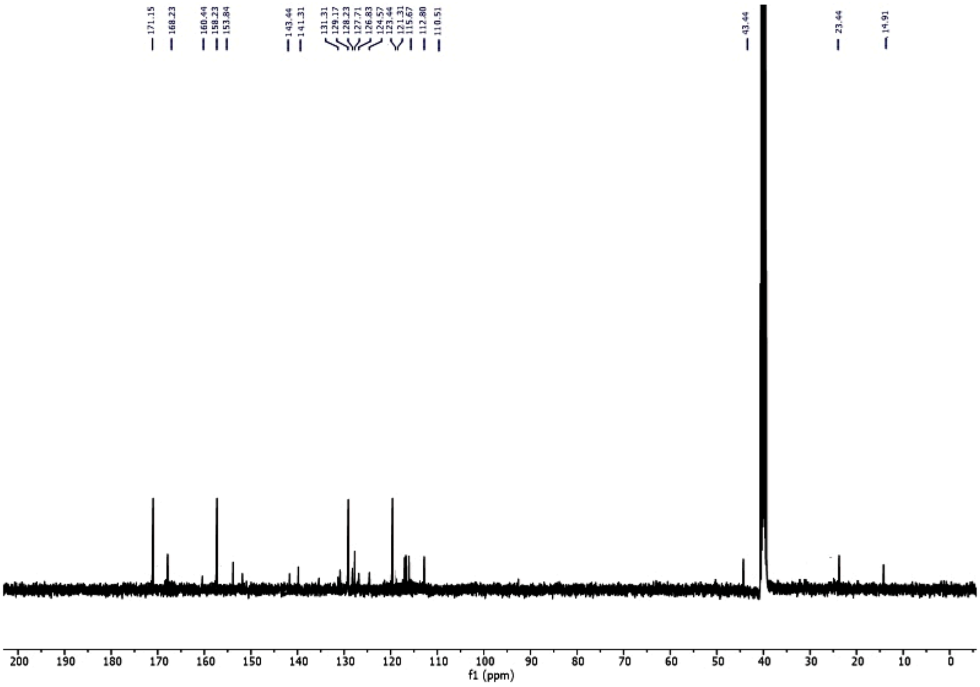

Figure S39. ^{13}CNMR

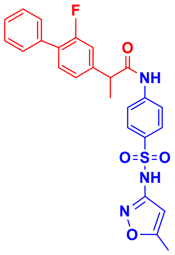

Figure S40. 2-(2-fluoro-[1,1′-biphenyl]-4-yl)-N-(4-(N-(5-methylisoxazol-3-yl)sulfamoyl)phenyl)propanamide (**17**)

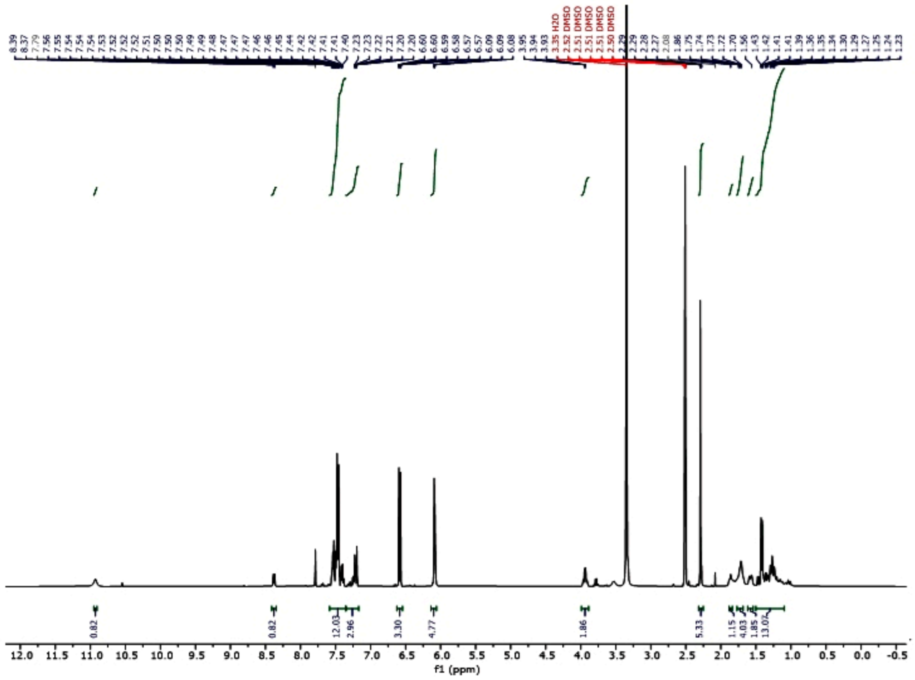

Figure S41. ^{1}HNMR

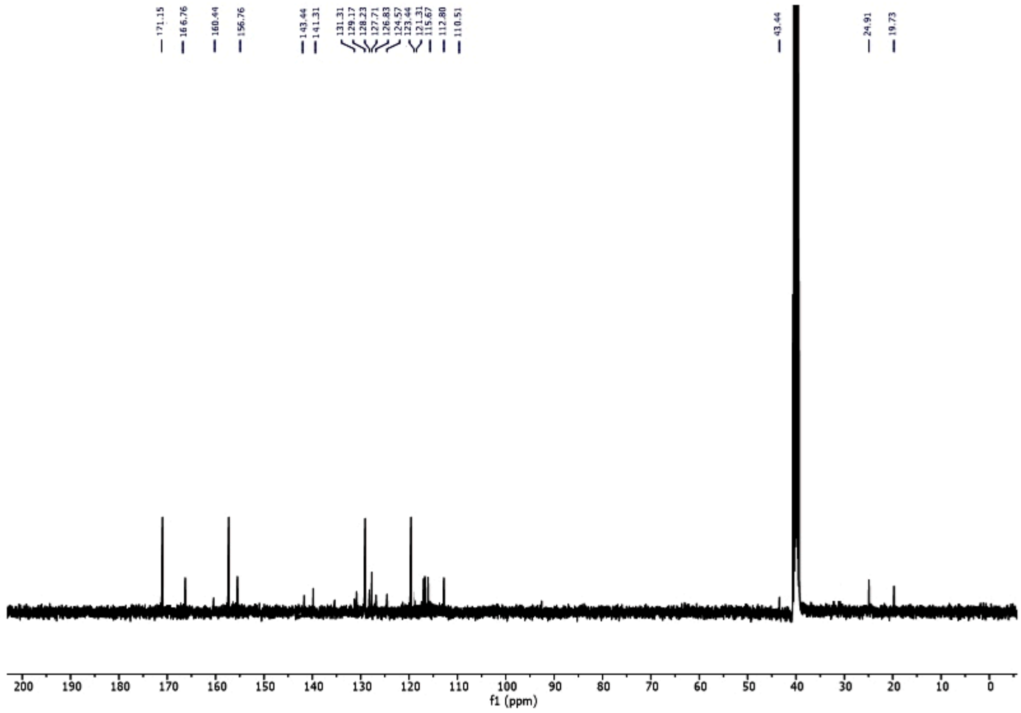

Figure S42. ^{13}CNMR

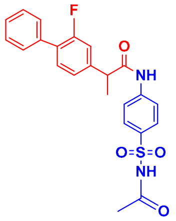

Figure S43. N-(4-(N-acetylsulfamoyl)phenyl)-2-(2-fluoro-[1,1′-biphenyl]-4-yl)propanamide (**18**)

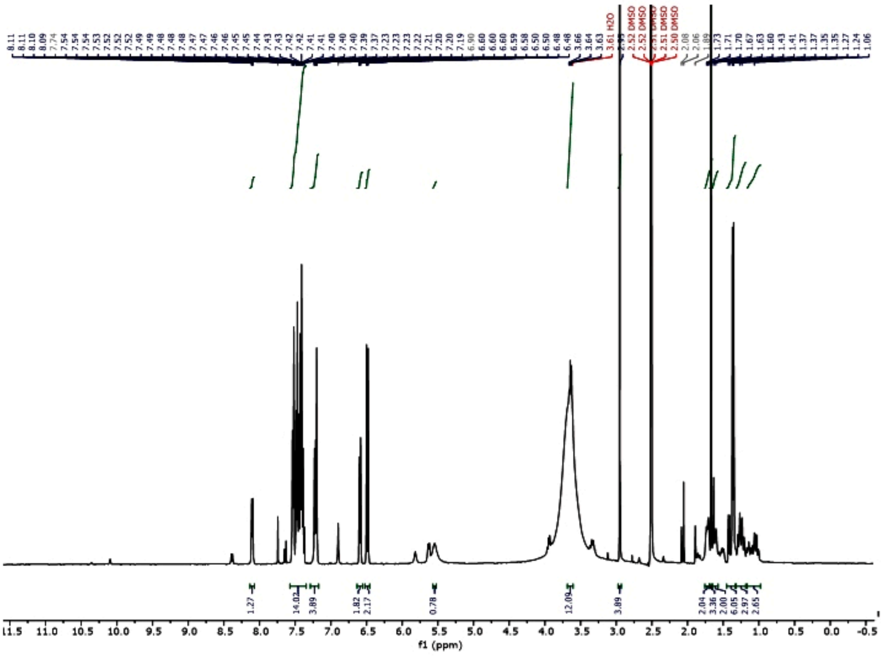

Figure S44. ^{1}HNMR

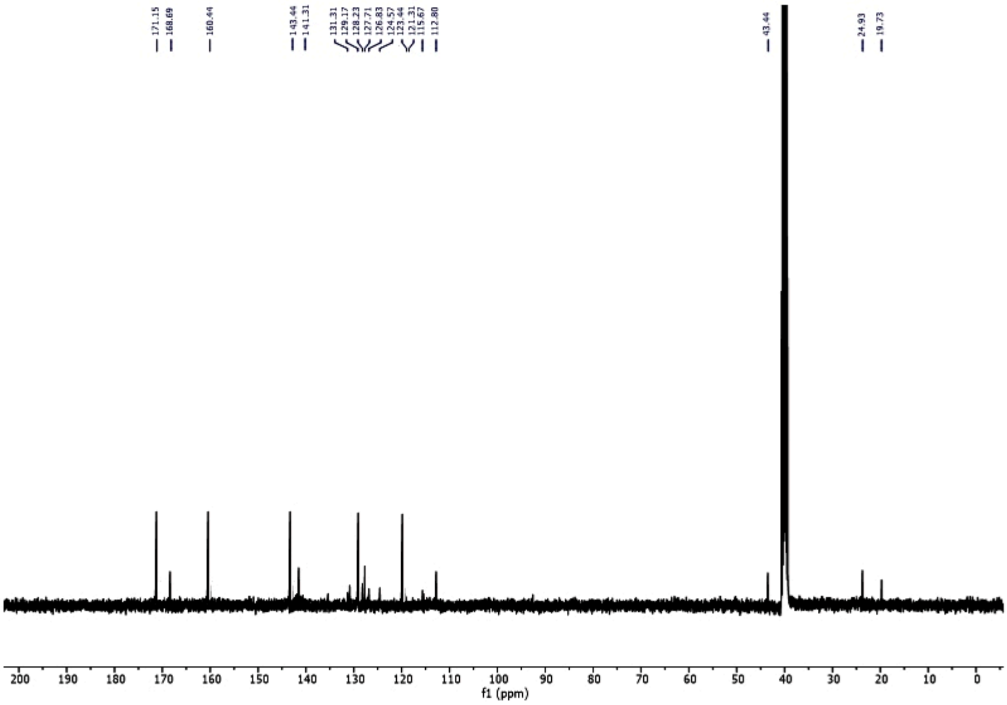

Figure S45. ^{13}CNMR

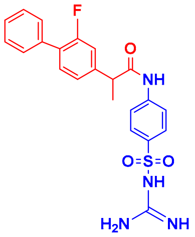

Figure S46. N-(4-(N-carbamimidoylsulfamoyl)phenyl)-2-(2-fluoro-[1,1′-biphenyl]-4-yl)propanamide (**19**)

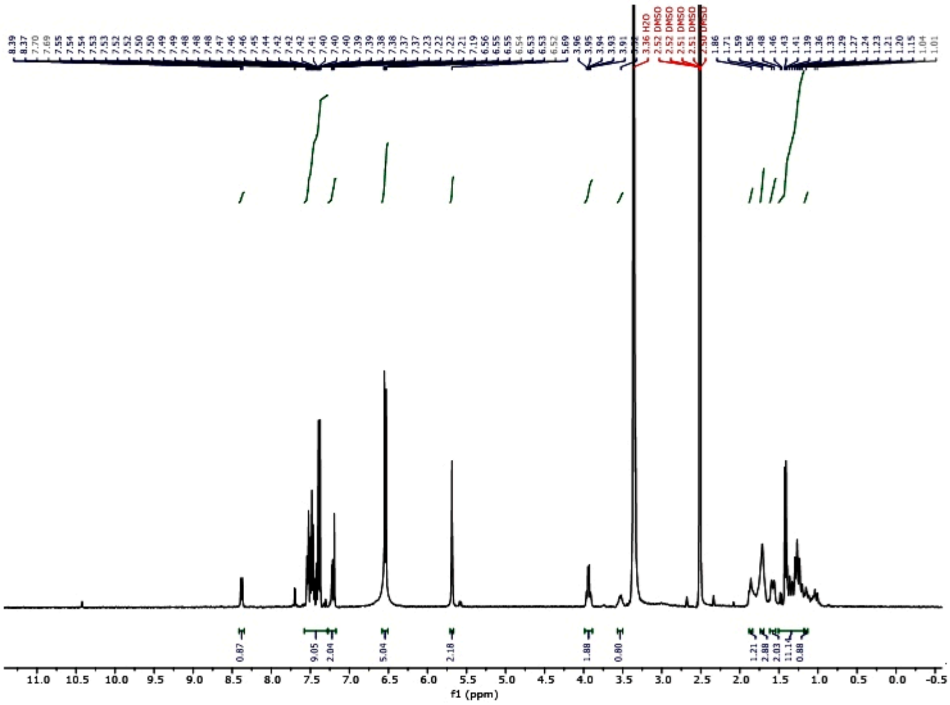

Figure S47. ^{1}HNMR

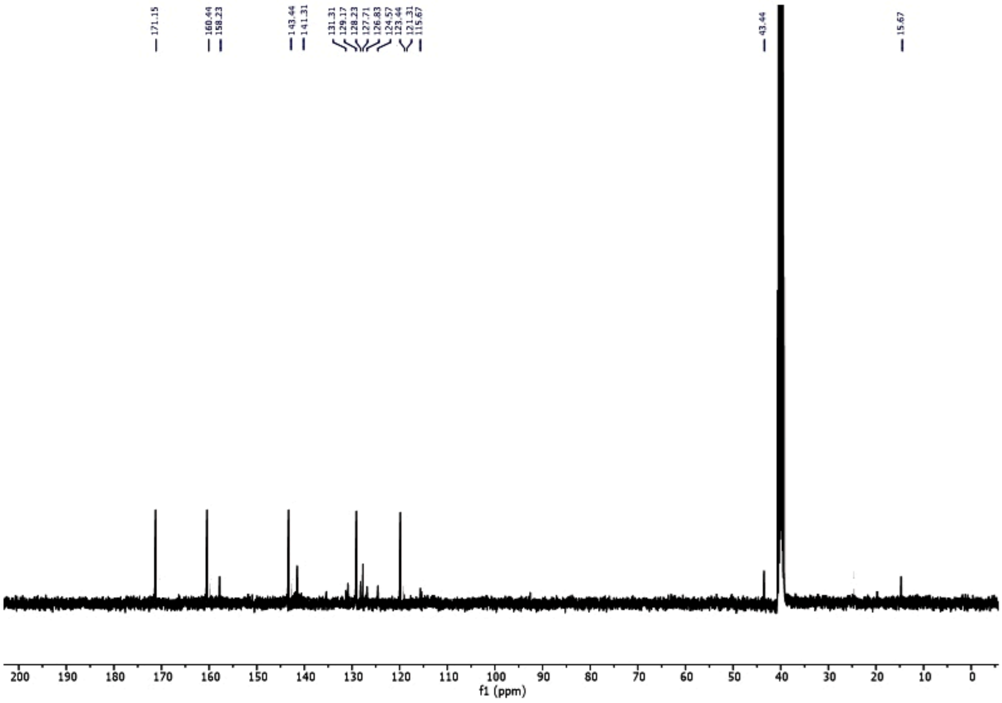

Figure S48. ^{13}CNMR
